# Genetic sharing and heritability of paediatric age of onset autoimmune diseases

**DOI:** 10.1038/ncomms9442

**Published:** 2015-10-09

**Authors:** Yun R. Li, Sihai D. Zhao, Jin Li, Jonathan P. Bradfield, Maede Mohebnasab, Laura Steel, Julie Kobie, Debra J. Abrams, Frank D. Mentch, Joseph T. Glessner, Yiran Guo, Zhi Wei, John J. Connolly, Christopher J. Cardinale, Marina Bakay, Dong Li, S. Melkorka Maggadottir, Kelly A. Thomas, Haijun Qui, Rosetta M. Chiavacci, Cecilia E. Kim, Fengxiang Wang, James Snyder, Berit Flatø, Øystein Førre, Lee A. Denson, Susan D. Thompson, Mara L. Becker, Stephen L. Guthery, Anna Latiano, Elena Perez, Elena Resnick, Caterina Strisciuglio, Annamaria Staiano, Erasmo Miele, Mark S. Silverberg, Benedicte A. Lie, Marilynn Punaro, Richard K. Russell, David C. Wilson, Marla C. Dubinsky, Dimitri S. Monos, Vito Annese, Jane E. Munro, Carol Wise, Helen Chapel, Charlotte Cunningham-Rundles, Jordan S. Orange, Edward M. Behrens, Kathleen E. Sullivan, Subra Kugathasan, Anne M. Griffiths, Jack Satsangi, Struan F. A. Grant, Patrick M. A. Sleiman, Terri H. Finkel, Constantin Polychronakos, Robert N. Baldassano, Eline T. Luning Prak, Justine A. Ellis, Hongzhe Li, Brendan J. Keating, Hakon Hakonarson

**Affiliations:** 1 Center for Applied Genomics, Children's Hospital of Philadelphia, Philadelphia, Pennsylvania 19104, USA; 2 Medical Scientist Training Program, Perelman School of Medicine, University of Pennsylvania, Philadelphia, Pennsylvania 19104, USA; 3 Department of Statistics, University of Illinois at Urbana-Champaign, Champaign, Illinois 61820, USA; 4 Department of Biostatistics and Epidemiology, Perelman School of Medicine, University of Pennsylvania, Philadelphia, Pennsylvania 19104, USA; 5 Department of Computer Science, New Jersey Institute of Technology, Newark, New Jersey 07103, USA; 6 Division of Allergy and Immunology, Children's Hospital of Philadelphia, Philadelphia, Pennsylvania 19104, USA; 7 Department of Rheumatology, Oslo University Hospital, Rikshospitalet, Oslo 0372, Norway; 8 Center for Inflammatory Bowel Disease, Division of Gastroenterology, Cincinnati Children's Hospital Medical Center, Cincinnati, Ohio 45229, USA; 9 Divison of Rheumatology, Cincinnati Children's Hospital Medical Center, Cincinnati, Ohio 45229, USA; 10 Division of Rheumatology and Division of Clinical Pharmacology, Toxicology, and Therapeutic Innovation, Children's Mercy-Kansas City, Kansas City, Missouri 64108, USA; 11 Department of Pediatrics, University of Utah School of Medicine and Primary Children's Medical Center, Salt Lake City, Utah 84113, USA; 12 RCCS ‘Casa Sollievo della Sofferenza', Division of Gastroenterology, San Giovanni Rotondo 71013, Italy; 13 Division of Pediatric Allergy and Immunology, University of Miami Miller School of Medicine, Miami, Florida 33136, USA; 14 Institute of Immunology, Department of Medicine, Icahn School of Medicine at Mount Sinai, Mount Sinai Hospital, New York, New York 10029, USA; 15 Department of Translational Medical Science, Section of Pediatrics, University of Naples "Federico II", Naples 80138, Italy; 16 IBD Centre, Mount Sinai Hospital, University of Toronto, 441-600 University Avenue, Toronto, Ontario, Canada M5G 1X5; 17 Department of Immunology, Oslo University Hospital, Rikshospitalet, 0027 Oslo 0372, Norway; 18 Texas Scottish Rite Hospital for Children, Dallas, Texas 750219, USA; 19 Yorkhill Hospital for Sick Children, Glasgow G38SJ, Scotland; 20 Paediatric Gastroenterology and Nutrition, Royal Hospital for Sick Children, Edinburgh and Child Life and Health, University of Edinburgh, Edinburgh EH9 1UW, UK; 21 Departments of Pediatrics and Common Disease Genetics, Cedars Sinai Medical Center, Los Angeles, California 90048, USA; 22 Department of Pathology, Children's Hospital of Philadelphia, Philadelphia, Pennsylvania 19104, USA; 23 Department of Pediatrics, The Perelman School of Medicine, University of Pennsylvania, Philadelphia, Pennsylvania 19104, USA; 24 Unit of Gastroenterology, Department of Medical and Surgical Specialties, Careggi University Hospital, Viale Pieraccini 18, Florence 50139, Italy; 25 Paediatric Rheumatology Unit, Royal Children's Hospital, Parkville, Victoria 3052, Australia; 26 Arthritis and Rheumatology Research, Murdoch Childrens Research Institute, Parkville, Victoria 3052, Australia; 27 Sarah M. and Charles E. Seay Center for Musculoskeletal Research, Texas Scottish Rite Hospital for Children, Dallas, Texas 750219, USA; 28 Department of Clinical Immunology, Nuffield Department of Medicine, University of Oxford, OX1 1NF, UK; 29 Section of Immunology, Allergy, and Rheumatology, Department of Pediatric Medicine, Texas Children's Hospital, Houston, Texas 77030, USA; 30 Division of Rheumatology, Children's Hospital of Philadelphia, Philadelphia, Pennsylvania 19104, USA; 31 Department of Pediatrics, Emory University School of Medicine and Children's Health Care of Atlanta, Atlanta, Georgia 30329, USA; 32 Hospital for Sick Children, University of Toronto, 555 University Avenue, Toronto, Ontario, Canada M5G 1X8; 33 Gastrointestinal Unit, Division of Medical Sciences, School of Molecular and Clinical Medicine, University of Edinburgh, Western General Hospital, Edinburgh EH4 2XU, UK; 34 Department of Pediatrics, Nemours Children's Hospital, Orlando, Florida 32827, USA; 35 Departments of Pediatrics and Human Genetics, McGill University, Montreal, Quebec, Canada H3H 1P3; 36 Division of Gastroenterology, Children's Hospital of Philadelphia, Philadelphia, Pennsylvania 19104, USA; 37 Department of Pathology and Lab Medicine, Perelman School of Medicine University of Pennsylvania, Philadelphia, Pennsylvania 19104, USA; 38 Genes, Environment and Complex Disease, Murdoch Childrens Research Institute, Parkville, Victoria 3052, Australia; 39 Department of Paediatrics, University of Melbourne, Parkville, Victoria 3052, Australia; 40 Division of Pulmonary Medicine, Children's Hospital of Philadelphia, Philadelphia, Pennsylvania 19104, USA

## Abstract

Autoimmune diseases (AIDs) are polygenic diseases affecting 7–10% of the population in the Western Hemisphere with few effective therapies. Here, we quantify the heritability of paediatric AIDs (pAIDs), including JIA, SLE, CEL, T1D, UC, CD, PS, SPA and CVID, attributable to common genomic variations (SNP*-h*^*2*^). SNP-*h*^*2*^ estimates are most significant for T1D (0.863±s.e. 0.07) and JIA (0.727±s.e. 0.037), more modest for UC (0.386±s.e. 0.04) and CD (0.454±0.025), largely consistent with population estimates and are generally greater than that previously reported by adult GWAS. On pairwise analysis, we observed that the diseases UC-CD (0.69±s.e. 0.07) and JIA-CVID (0.343±s.e. 0.13) are the most strongly correlated. Variations across the *MHC* strongly contribute to SNP*-h*^*2*^ in T1D and JIA, but does not significantly contribute to the pairwise *rG*. Together, our results partition contributions of shared versus disease-specific genomic variations to pAID heritability, identifying pAIDs with unexpected risk sharing, while recapitulating known associations between autoimmune diseases previously reported in adult cohorts.

## Abstract (web summary)

Autoimmune diseases are genetically complex disorders that affect up to 10% of the Western population. Here Li *et al*. quantify the heritability of a range of autoimmune diseases in the largest paediatric cohort examined to date, illustrating that genetic and non-genetic components variably contribute to the susceptibility of each disease.

## Graphical abstract

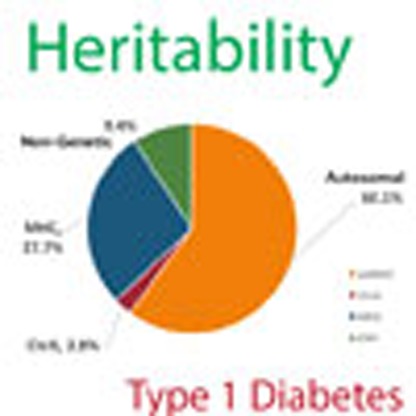

Autoimmune (AI) diseases affect approximately 1 in 12 individuals living in the Western Hemisphere, representing a significant cause of morbidity, chronic disability and health-care burden. High rates of sibling recurrence and twin–twin concordance, both within and across multiple independent AI diseases, coupled with recent results from genome-wide association studies (GWAS), suggest that a set of shared genetic risk factors underlie paediatric AI disease (pAID) aetiology^1^,^2^,^3^. Moreover, a number of AI diseases show clear familial clustering, such as inflammatory bowel disease (IBD)^4^, whereas others (for example, type 1 diabetes (T1D), AI thyroiditis (THY) and celiac disease (CEL) may manifest as comorbid diseases in polyglandular AI syndromes^2^. Although the concept of genetic sharing among AIs is intriguing, it remains unclear if this is due to ‘pleiotropic' risk factors that predispose to multiple AI diseases via shared mechanisms or if multiple, independent risk factors are responsible.

GWAS have identified single-nucleotide polymorphisms (SNPs) across hundreds of loci as being associated with an increased risk of developing AI^5^,^6^,^7^,^8^,^9^,^10^,^11^,^12^. These findings, coupled with those from epidemiological studies, strongly support the existence of (i) an overlapping ‘AI disease genetic landscape'^13^,^14^ and (ii), consequently, a shared heritability across these diseases. Heritability, in the broad-sense (*H*^*2*^), is defined as the entirety of an individual's phenotypic variation explained by genetic variance, but in practicality, it can be difficult to quantify and partition precisely^15^. A major contribution to *H*^*2*^ is the narrow-sense or additive heritability (*h*^*2*^), which can be more accurately quantified.^15^. Recently, a new method was established to estimate the total phenotype variance attributable to additive genetic variations using genome-wide SNP genotyping data^16^,^17^,^18^,^19^. The method has been since applied to dozens of GWAS-examined traits and extended to examine jointly the co-heritability of related diseases^20^.

We systematically quantified the narrow-sense heritability, *h*^*2*^, as well as the pairwise joint heritability of pAIDs attributable to common genomic variation using a single-centre accrued cohort of over 5,000 unrelated cases composed of nine independent pAIDs and 36,000 shared, population-based healthy controls. We first report the genome-wide SNP genotype-derived heritability estimates (referred to as SNP-*h*^*2*^) and then the genetic correlation (SNP-*rG*) across pairs of the nine investigated pAIDs. We contextualize these findings alongside a comprehensive review of available literature and epidemiological data sets, illustrate a method for quantifying genetic risk factor sharing across pAIDs, and provide considerations for how such genetic data can aid disease prediction.

## Results

### Quantifying the heritability of paediatric AI diseases

To quantify the SNP-*h*^*2*^ of the nine pAIDs, we utilized genome-wide SNP genotypes ascertained from DNA samples of patients of each pAID cohort along with samples from population-based control subjects with no known diagnosis of autoimmunity or immunodeficiency. Following extensive quality control (QC), removing SNPs of lower minor allele frequency (MAF), missingness and differential missingness in cases and controls, and deviation from Hardy–Weinberg equilibrium (see Methods), we retained 461,301 SNPs. We excluded samples for low genotyping rates, cryptic relatedness and genetic outliers, leaving a cohort consisting of 4,956 cases distributed across nine pAIDs and 27,451 unrelated shared population-based controls (Table 1). We also included, for comparison, a non-immune-mediated dichotomous trait, paediatric-onset epilepsy (EPI); this cohort of ∼800 case subjects was recruited and genotyped at our centre using the same platforms over the same time period.

**Figure 1:**
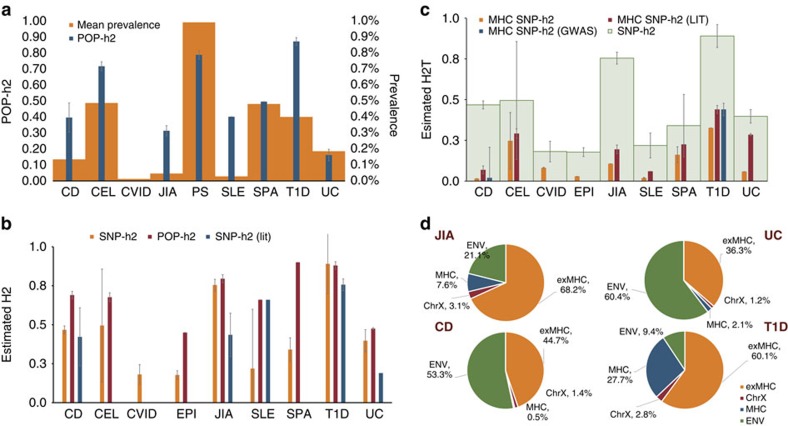
Autoimmune disease prevalence and heritability estimates. (**a**) Mean population-based AI disease prevalence (orange) and heritability (blue) estimates (mean±s.d.). Data are curated from epidemiological surveys among Caucasian populations in Europe or North America based on studies indexed in PubMed between 1975 and 2015. Where multiple sources of data are available for a given trait, we reported a simple non-weighted arithmetic mean and provided as error bars the standard deviation. Most heritability estimates were based on twin concordance rates. Raw data used and references can be found in Supplementary Tables 1 and 2. (**b**) Univariate SNP-heritability (SNP-*h*^*2*^, orange) compared with estimates reported by prior studies. (SNP-*h*^*2*^ (lit), blue) based on variations across the autosomes compared with population-based estimates (POP-*h*^*2*^, red) as reported in the literature (lit). Raw data used from prior GWAS SNP-*h*^*2*^ estimates are provided in Supplementary Table 3. Error bars denote standard error. (**c**) Univariate SNP-heritability (autosomal) estimates with (Light green, wide) and without the extended *MHC* (orange, narrow). Results are compared with corresponding heritability estimates reported using population-based (red, narrow) versus other published SNP-heritability estimates (blue, narrow), when available for a given disease. Literature data used and references can be found in Supplementary Table 2 and Supplementary Tables 6 and 7. Error bars denote standard error. (**d**) Partitioning phenotypic variance to genetic and non-genetic (ENV, green) components in the four largest pAID cohorts. Genetic components include contributions from the entire autosomal regions excluding the *MHC* (ex*MHC*, orange), the extended *MHC* (*MHC*, blue) alone as well as from the *X-chromosome* (*ChrX*, red).

We used a previously described method for estimating disease variance explained by additive genetic factors using GWAS data (referred to as SNP-based heritability or SNP-*h*^*2*^)^17^. We transformed the SNP-*h*^*2*^ estimates from the observed to the liability-scale using respective observed disease prevalence. To assess if our SNP-*h*^*2*^ estimates are consistent with previously published findings and other population-based heritability estimates (POP-*h*^*2*^), we performed a systematic literature search followed by manual curation of prevalence and heritability estimates for each of the nine pAIDs (Fig. 1a and Supplementary Tables 1 and 2).

Among the pAIDs examined where the SNP-*h*^*2*^ estimates were at least nominally significant (*P*<0.05), T1D and juvenile idiopathic arthritis (JIA) were the most highly heritable (Fig. 1b). Considerably lower estimates were observed for ulcerative colitis (UC) and Crohn's disease (CD; Supplementary Fig. 1A), suggesting that environmental factors may play a much larger role in IBD aetiology (Fig. 1d). We also observed relatively low SNP-*h*^*2*^ estimates for systemic lupus erythematosus (SLE; 0.205±s.e. 0.076).

### Contribution of the MHC region and *ChrX* to SNP-h^2^

Given the known association of variants across the *MHC* with AI diseases, we quantified their contribution to the SNP-*h*^*2*^ for each of the nine pAIDs. We first performed HLA imputation^21^, to identify the most strongly associated SNP, amino acid or HLA allele with each pAID (Supplementary Table 5) and we estimated POP-*h*^*2*^ attributable to the extended *MHC* based on previous analyses (Supplementary Tables 6 and 7). The *MHC*-specific SNP-*h*^*2*^ estimates correlated well with the strength of lead *MHC P*-value. For example, variations across the extended *MHC* region accounted for 32.7% of the total autosomal SNP-*h*^*2*^ in T1D and 24.7% of that in CEL, with no significant contribution to the SNP-*h*^*2*^ estimates in psoriasis (PS), SLE, CD or the non-pAID, EPI. Despite the pervasive association between SNPs within the *MHC* and both JIA and UC, contributions of the extended *MHC* to their total SNP-*h*^*2*^ (10.7% and 5.8%, respectively) were limited (Fig. 1c and Table 2). Despite the known association with HLA-DRB1*0103 and HLA-B*52 in UC^13^, we observed that removing the extended *MHC* did not significantly reduce the observed SNP-*h*^*2*^ for either UC or, the related IBD phenotype, CD (Supplementary Table 8). As expected, the contribution of *ChrX* to the overall SNP-*h*^*2*^ was small across all pAIDs (Supplementary Table 2). These estimates are consistent with expectations as *ChrX* makes up only about 5% of the total genome^22^, has comparatively fewer coding bases and is less polymorphic^23^.

### Disease prediction using support vector machines (SVM)s

Given that we observed relatively high rates of heritability across many of the pAIDs, we evaluated the utility of common genomic variations in predicting pAID disease risk, using a SVM model-based approach. Using a tenfold cross-validation study design, we built a linear SVM model using the top GWAS signals observed using nine out of ten of the total samples and tested this SVM predictor in the remaining 10% of the samples. Based on previous analyses in both case–control^24^ and quantitative traits^25^, we expect that disease prediction accuracy to behave as function of heritability, sample size and the number of causal variants. We assessed the mean and maximum area under the receiver operating characteristic curve (AUC) achieved, showing that our SVM predictor was most effective for JIA and T1D (AUC_max_>0.9; AUC_mean_>0.85), although satisfactory results was also seen in CEL (AUC_max_>0.8 and AUC_mean_>0.7). These findings are consistent with that recently reported by Speed *et al*. using an independent adult CEL cohort^26^. The predictability of all nine pAIDs was fairly robust to range of *P*-value thresholds used for selecting SNP predictors in building the SVM model (Fig. 3 and Supplementary Table 11).

### Estimation of pairwise co-heritability across pAIDs

**Figure 2:**
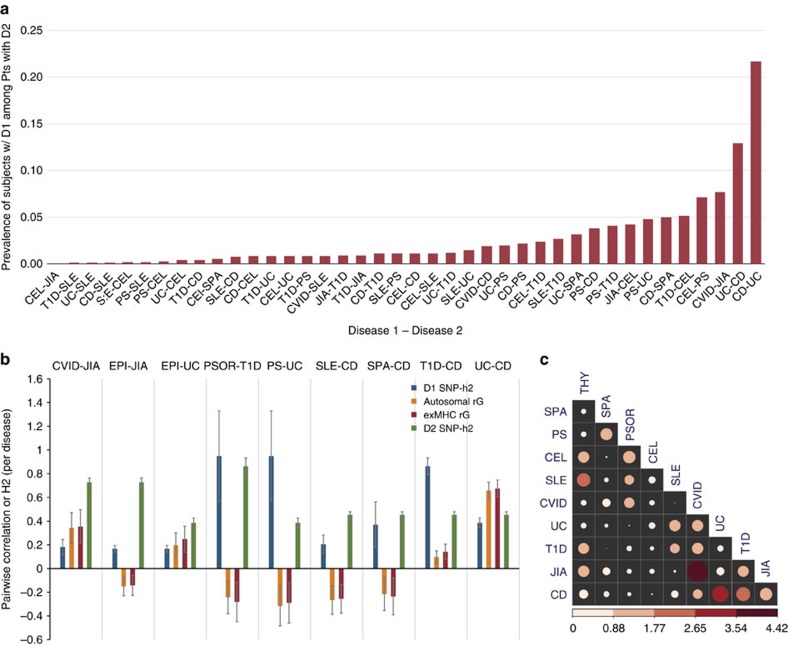
Prevalence of AI disease co-morbidities and estimates of genetic correlation (co-heritability) across pAIDs. (**a**) Observed prevalence of pAID comorbidity observed in Caucasian populations in Europe and North America as curated from large-scale cohort studies. For each pairwise combination (for example, Disease 1–Disease 2), the rate (*y* axis) indicates the percentage of patients with Disease 2 who have also been diagnosed with Disease 1. Literature data used and references can be found in Supplementary Table 9. (**b**) Bivariate estimates of genetic correlation (pairwise co-heritability) across pAIDs. The heritability (SNP-*h*^*2*^) for the first and second disease are shown for each pAID pair (blue and green bars, respectively) along with the genetic correlation (*rG*) for the pair estimated based on total autosomal common genetic variants (orange) and based on autosomal variants excluding the *MHC* (red). Displayed are those pairs for which the *rG* estimates reached nominal significance (*P*<0.05). *P*-values are based on restricted maximum likelihood ratio test. Error bars represent standard error. (**c**) Genetic sharing using the genome-wide pairwise sharing statistic (GPS). Correlation plot of the *P*-values obtained from the genome-wide pairwise shared analysis. Significant *P*-values support evidence of genetic sharing based on the correlation of significant association findings reported by GWAS for each pair of diseases.

To investigate diseases with shared underlying genetic risk factors, we assessed the genetic correlation *(rG)* for each pair of pAIDs and between each of the nine pAIDs and EPI, which provided a comparative baseline for non-significant genetic correlation^20^. We used both a strict (*P*_BS_) and a more relaxed Bonferroni correction (*P*_BL_) to adjust for either 45 (all pairwise combinations) or 9 comparisons (combinations per pAID); (see Methods). We observed the highest *rG* between UC and CD (*rG*=+0.66; *P*_BS_<0.001), consistent with the reported sharing of association loci by several published GWAS, immunochip and fine-mapping studies^11^,^27^,^28^,^29^ (Supplementary Table 10). We also noted a positive *rG* between common variable immunodeficiency disorder (CVID) and JIA (*rG*=+0.34), although it was more modest (*P*_BL_<0.01). While we did observe a marginally positive *rG* for CD and T1D consistent with results from published GWAS metanalysis^30^, although it did not reach significance at a liberal Bonferroni threshold (*rG*=+0.096; *P*_BL_=0.17). Of note, we did not observe a significant reduction in *rG* estimates when the extended *MHC* was entirely removed from the analysis across any of the pAID pairs, making it unlikely that the sharing of common *HLA* alleles could significantly account for the degree of co-heritability observed (Fig. 2b).

## Discussion

To our knowledge, this is the most comprehensive assessment of heritability and disease prediction using genome-wide dense genotyping data across multiple pAIDs. The results show that SNP-*h*^*2*^ estimates were significantly higher for the pAID cohorts as compared with those obtained for the non-immune-mediated disease EPI (Fig. 1a and Supplementary Tables 1 and 2). Among the pAIDs examined where the SNP-*h*^*2*^ estimates were at least nominally significant (*P*<0.05), T1D and JIA were the most highly heritable (Fig. 1b). These results are in keeping with the SNP-*h*^*2*^ estimates reported for T1D and Rheumatoid Factor Positive (RF+), Rheumatoid Arthritis (RA) in adults, using the Wellcome Trust Case Control Consortium data sets^17^,^26^,^31^. Considerably weaker SNP-*h*^*2*^ estimates were observed for UC and CD, consistent with previous reports in adults^32^ (Supplementary Fig. 1A). Although the sample size of CD was several fold greater than those of T1D and UC, and twice that for JIA, the SNP-*h*^*2*^ estimates are lower in CD, suggesting that environmental factors play a much larger role in CD disease aetiology (Fig. 1d). This finding is in keeping with studies demonstrating a key role for the gut microbiome and faecal flora in disease-onset and severity in the IBDs^11^,^33^,^34^.

As noted, the SNP-*h*^*2*^ observed for JIA was high despite the known heterogeneous nature of this disease, including seven distinct JIA subtypes^35^. Little is known about the heritability of JIA as it is fairly uncommon. However, in RA, the more common JIA counterpart in adults, a range of SNP-*h*^*2*^ estimates has been reported^17^,^26^,^31^,^36^. Some of the heterogeneity in SNP-*h*^*2*^ estimates for RA may be attributable to the different ratios of RF+ vs RF− patients across different study cohorts, as recent analyses suggest that RF+ RA may be ‘distinct' from RF− forms of RA in terms of genetic aetiology^37^. Moreover, the subphenotype of JIA that is most similar to RF+ RA (i.e. RF+ JIA) made up only a small component of our JIA cohort (4.9%). Thus, the high estimated heritability observed in JIA suggests that despite the heterogeneous clinical findings, there may be a strongly shared genetic component contributing to a common aetiology.

We observed relatively low SNP-*h*^*2*^ estimates for SLE (Fig. 1b). Although these estimates are lower than those reported by *So et al*.,^38^,^39^ they are higher than the POP-*h2* reported based on sibling-recurrence^40^. These observations are consistent with strong environmental and epigenetic components to SLE liability^41^,^42^. We included in our analysis a non-immune-mediated disease, early-onset EPI, as a comparator cohort. As expected, the SNP-*h*^*2*^ estimates on the liability scale, albeit non-zero, was relatively low compared with any of the AI diseases. That we observed slightly higher heritability estimates across our paediatric cohorts than previously reported in adults is also in keeping with the notion that paediatric-onset diseases have been noted previously to reflect disease aetiologies with a stronger genetic component^29^ and less confounding due to reduced timespan of environmental exposure(s). Adult or late-onset AI diseases can be associated with environmental precipitating factors such as viral infections or drug exposures, which have been implicated in a range of AI diseases including T1D, CEL and SLE^3^,^42^.

Although estimates for JIA and T1D are higher than SNP-*h*^*2*^ estimates reported previously, our estimates for RA and T1D are more consistent, although still falling short of, than those reported by population estimates from twin-based or familial studies (Supplementary Table 2). That these SNP-h2 based estimates are in general still falling behind estimates made from epidemiological studies illustrates the ‘missing heritability' phenomenon. Disparities between POP-*h*^*2*^ and SNP-*h*^*2*^ estimates may be at least partially attributable to inflation of population-based estimates in the presence of ascertainment-bias and/or insufficient adjustment for confounding effects. The latter tends to occur if there are significant non-additive or shared environmental factors that contribute to phenotypic variation^36^,^43^.

A number of previous epidemiological and genetic studies have suggested a significant degree of shared risk across AI diseases^44^,^45^,^46^,^47^. There are a number of reasons why our results may differ from these reports. In such population-based studies, observed sharing of risk in the population is inevitably confounded by common environmental factors or gene–environment interactions, neither of which would be parsed out from purely epidemiological observations. In addition, it can be challenging to perform these comparisons in heterogeneous populations because they may be composed of different underlying genetic backgrounds, and genetic ancestry is known to dramatically affect the risk for many AI diseases (for example, greater risk of CEL and JIA in Caucasians)^4^,^20^.

Although there are several prior large-scale analyses of genetic sharing among AI diseases using GWAS data, these are based on somewhat different analytical approaches or study methodology than those employed here. A notable example comes from Cotsapas *et al*., who derived a Cross-Phenotype Meta-Analysis test statistic that powerfully combines multiple independent AI data sets to analyse the likelihood that a SNP is shared across disease phenotypes. They applied this test statistic to the 140 top genetic risk variants reported previously by GWAS across seven AI diseases^47^. Although there is no doubt that findings from this study are informative, the targeted candidate approach has clear limitations and only summary statistics were available. Another concern, which is not unique to the study by Cotsapas *et al*., but a concern in most large GWAS meta-analyses, is inter-study heterogeneity these studies often combine summary data obtained from independent case–control study cohorts accrued and genotyped across North America and Europe using different genotyping platforms and QC/analysis steps, requiring *post-hoc* statistical adjustments for heterogeneity, genetic variation and the use of SNP proxies. Although single-institution study designs can have limited applicability, in our study, using a common shared control accrued in the same institution and genotyped on the same platform does limit the effect of inter-study heterogeneity in our analysis.

As expected, we found that variations across the extended *MHC* strongly contributed to both heritability estimates and disease risk predictability in T1D and CEL, and more modestly in UC and JIA. The contribution of the extended *MHC* to total phenotypic variance explained correlated with the strength of the strongest association signal within the extended *MHC*. However, as recent reports have shown, this method for estimating *h*^*2*^ is sensitive to the variation in linkage-disequilibrium (LD) across the genome^18^,^31^. We therefore examined the effect of LD on the SNP-*h*^*2*^ estimates by comparing the results with those obtained using non-correlated SNP markers (Supplementary Fig. 2B; see Methods for details). As anticipated, the effect of the pruning is mostly attributable to the strong role of the *MHC* in the heritability of these diseases, as pruning had little effect on the heritability estimates once the extended *MHC* was removed. Thus, the number and degree of LD for the input SNPs used for calculating *h*^*2*^ can be important for diseases where the *MHC* plays a major role, consistent with previous studies^31^,^48^,^49^.

The SNP-*h*^*2*^ for T1D was most strongly affected by the removal of the extended *MHC*, emphasizing the importance of *MHC* polymorphisms in T1D pathogenesis. In addition, the estimates for SPA and CEL both fell significantly when markers across the *MHC* were excluded from further analysis. The relatively limited contribution of the genetic polymorphisms across the *MHC* to heritability in IBD was consistent with prior GWAS results, as the *MHC* SNP (rs1626392, *P*<2.27 × 10^−7^) most significantly associated with CD did not reach genome-wide significance, defined as *P*<5 × 10^−8^ (Supplementary Table 8). Aside from the MHC, recent work has examined the degree to which functional or coding loci, for example, DNAse I Hypersensitivity Sites^50^, contribute to disease heritability. Such studies, currently underway, will help delineate biological functions and connect genetic associations with mechanistic roles of such functional variants.

**Figure 3:**
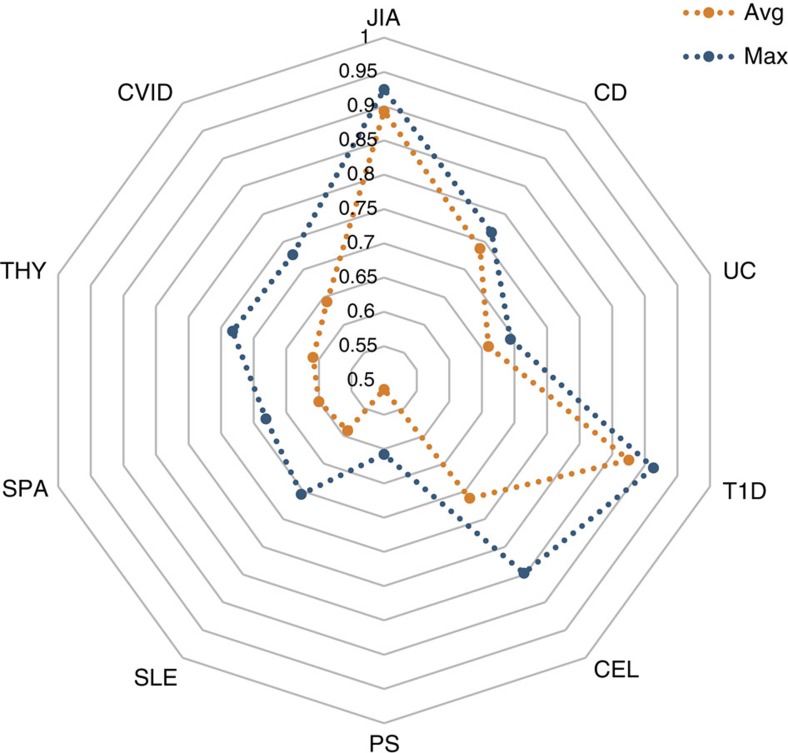
Disease prediction using a support vector machine model. Shown are the mean (orange) and maximum (blue) areas under the curve (AUC) achieved in the validation set as obtained for each disease in the ten-fold cross-validation analysis. The mean and maxima refer to the best AUC's when testing a range of *P*-value thresholds from which to pick SNPs in training the linear SVM. SPA, spondyloarthropathy.

A still unrealized, but much anticipated goal of personalized medicine is to utilize genomic data to accurately predict disease risk^26^,^51^,^52^,^53^. We found that for the three pAIDs (T1D, JIA and CEL) that were most predictable, a range of *P*-value thresholds (*P*<1 × 10^−6^ and *P<*1 × 10^−8^) could be used to identify the predictive SNPs without significantly impacting maximum or mean AUC achieved, suggesting that the SVM model was robust to this parameter (Fig. 3 and Supplementary Table 11). In comparison, we obtained fairly modest AUCs for CD, UC and CVID (AUC_max_>0.7, AUC_mean_>0.65). These are in keeping with our expectation that genetic prediction should rest on underlying genetic heritability and confirms the value of SNP heritability analysis.

Indeed, the above observations are perhaps not surprising, given recent findings that support a strong contribution for environmental factors in disease susceptibility. For example, host-microbial interactions have been implicated in the pathogenesis of IBD and RA^11^,^54^. Furthermore, in CVID, it is well-established that although genetic risk factors play a role in disease risk, there is significant within-disease heterogeneity in terms of aetiology. Patients with CVID are often diagnosed in late adolescence, suggesting that environmental risk factors play a greater role. Likewise, most cases of paediatric-onset IBD also have a post-pubescent age of onset. This is in contrast to T1D, JIA or CEL, which are commonly diagnosed by or before the age of 12 years, although some degree of variability is observed. This is consistent with the correlations noted above, in that the three diseases with more moderate SNP-*h*^*2*^ estimates were also less predictable.

Among the three largest cohorts, namely JIA, UC and CD, CD was by far the largest. However, the heritability estimated for CD in our data set was the lowest of the three. As we know from prior studies that disease prediction is a function of heritability, sample size and the number of causal variants, we might expect the accuracy of disease prediction for CD to be relatively poor. This is exactly what we observed. In contrast, we had somewhat limited sample sizes for SPA, PS and CEL cohorts, and we caution against the interpretation of the high heritability estimates observed for PS. Another limitation of the present study is that we have not considered the role of rare, or potentially *de novo*, variants in the overall estimates of genetic heritability. As more sequencing data using either whole-exome or whole-genome approaches become available, future studies will help address this question.

A unique opportunity provided by our cohort was the ability to quantify pairwise pAID genetic correlations as numerous epidemiological analyses have shown that subsets of pAIDs co-cluster in families or exhibit high rates of comorbidity^55^,^56^,^57^ (Table 3). As pAID co-heritability has not been systematically examined using genome-wide SNP data, we aimed to identify pAIDs showing significantly positive *rG* (that are consequently co-heritable) versus diseases that are either genetically unrelated or negatively correlated (and are consequently ‘mutually-protective'). We calculated the *rG* for each pAID pair and between each of the nine diseases and EPI. This latter analysis provides a ‘control' or contextual baseline, akin to the inclusion of CD as a ‘null comparator' phenotype by the Psychiatric Genomics Consortium^20^. We observed a strongly and moderately positive *rG* between two pAID pairs, namely UC-CD and JIA-CVID.

Although the *MHC* made major contributions to the disease-specific heritability, we found no evidence that variations across the *MHC* significantly contributed to the pAID co-heritability for any of the investigated disease pairs (Fig. 2b). For the pAID pairs with significantly positive *rG*'s (UC-CD and JIA-CVID), we did not observe a significant reduction in *rG* estimates when the SNPs within the *MHC* were removed from the analysis, making it unlikely that genetic sharing of *MHC* haplotypes can explain the genetic correlation observed among pAIDs in this data set (Fig. 2b). In addition, that the UC-CD and JIA-CVID pairs were the two with the largest positive rG is also consistent with results we obtained using an independent genome-wide pairwise sharing metric for genetic correlation, in which we considered all genome-wide SNP markers except those within the extended *MHC* locus (Fig. 2c, see Methods for details). Although it may appear to be surprising given the known association with the *MHC* across all pAIDs, these results are in keeping with our finding that the most significant *MHC* association signals identified for each pAID was disease-specific and did not overlap across the nine pAIDs (Supplementary Table 5).

Somewhat unexpectedly, we observed a negative marginal *rG* across several pAID pairs, including SLE-CD, SPA-CD, PS-UC and PS-T1D. Although none of these was significant following a Bonferroni correction, in each of the negatively correlated pAID pairs, one of the two diseases is considered a ‘classic autoimmune' (that is, SLE, UC, and T1D), whereas the other pAID in the pair (that is, CD and PS) has been noted to have a strong ‘inflammatory' component.

Taken together, we report genome-wide SNP genotype-derived heritability estimates and genetic correlations of disease liability across pairs of nine investigated pAIDs using common and low-frequency genetic variants. We contextualized these findings alongside a comprehensive review of available literature and epidemiological data sets, illustrate a method for quantifying genetic risk factor sharing across pAIDs and provide considerations for how such genetic data can aid in disease prediction. We observed that SNP-*h*^*2*^ estimates in pediatric AI diseases tend to be greater in magnitude when compared to SNP-*h*^*2*^ reported previously based on GWAS data from studies of adult AI disease cohorts, particularly for T1D, UC, JIA/RA. Moreover, we also observed that the ‘co-heritability' across pAIDs was minimally attributable to shared *MHC* variations. While genomic screening in the general population on a large scale is not currently feasible, or of high utility (given the low disease prevalence and consequently, limited positive predictive value as well as the limitations in interpretability), our analysis suggests that there is a high heritability and disease predictability across the pAIDs. Future studies in larger sample sizes and in adult cohorts will be helpful in validating these results and developing new and improved methods for genome-based disease prediction and for the development of novel biomarkers that can be used to predict pAID risk.

## Methods

### Study population

Information regarding the patient cohorts have been published previously and are summarized briefly below.

Cases and controls were either directly ascertained as described in prior studies^29^,^53^,^58^,^59^,^60^,^61^,^62^,^63^,^64^,^65^ or obtained from de-identified samples and associated electronic medical records (EMRs) residing in the genomics biorepository at the Children's Hospital of Philadelphia. EMR searches were conducted using previously described algorithms^58^,^59^ based on phenotype mapping established using PheWAS ICD-9 code mapping tables^53^,^58^,^60^ in consultation with qualified physician specialists for each disease cohort. All DNA samples were assessed for QC and genotyped on the Illumina HumanHap550 or HumanHap610 platforms at the Center for Applied Genomics (CAG) at the Children's Hospital of Philadelphia (CHOP, Philadelphia, Pennsylvania, USA). Note that the patient counts below refer to the total recruited sample size from which we excluded non-qualified samples/genotypes that did not pass QC criteria required for inclusion in the genetic analysis (for example, because of relatedness or poor genotyping rate; see details below).

The IBD cohort comprised 2,796 individuals aged 2–17 years of European ancestry with biopsy-proven disease, including 1,931 with CD and 865 with UC, excluding all patients with unclassified type (IBD-U). Affected individuals were recruited from multiple centres from four geographically discrete countries and diagnosed before their nineteenth birthday according to the standard IBD diagnostic criteria, as previously reported^3^,^29^.

The T1D cohort consisted of 1,120 cases from nuclear family trios (one affected child and two parents), including 267 independent Canadian T1D cases collected in paediatric diabetes clinics in Montreal, Toronto, Ottawa and Winnipeg (Canada) and 203 T1D cases recruited at CHOP since September 2006. All patients were Caucasians by self-report and ranged in age between 3 and 17 years, with 7.9 years being the median age at onset. All patients have been treated with insulin since diagnosis. Disease diagnosis was based on these clinical criteria, rather than any laboratory tests.

The JIA cohort was recruited in the United States of America, Australia and Norway and comprised of a total of 1,123 patients with onset of arthritis at <16 years of age. JIA diagnosis and JIA subtype were determined according to the International League of Associations for Rheumatology revised criteria^35^ and confirmed using the JIA Calculator software^66^ (http://www.jra-research.org/JIAcalc/), an algorithm-based tool adapted from the International League of Associations for Rheumatology criteria. Before standard QC procedures and exclusion of non-European ancestry, the JIA cohort was comprised of 464 case subjects from Texas Scottish Rite Hospital for Children (Dallas, Texas, USA) and the Children's Mercy Hospitals and Clinics (Kansas City, Missouri, USA) of self-reported European ancestry; 196 subjects from the CHOP; 221 subjects from the Murdoch Childrens Research Institute (Royal Children's Hospital, Melbourne, Australia) and 504 subjects from the Oslo University Hospital (Oslo, Norway).

The CVID study population consisted of 223 patients from the Mount Sinai School of Medicine (New York City, New York, USA); 76 patients from the University of Oxford (London, England); 47 patients from the CHOP and 27 patients from the University of South Florida (Tampa, Florida). The diagnosis in each case was validated against the ESID/PAGID diagnostic criteria, as previously described^67^. Although the diagnosis of CVID is most commonly made in young adults (aged 20–40 years), all of the CHOP and University of South Florida cases had paediatric age of onset disease, whereas the majority of the cases from the Mount Sinai School of Medicine and Oxford had onset in young adulthood. We note that as the number of individuals with adult-onset CVID disease is so small (less than 5% of all cases presented), and all ten diseases have paediatric age of onset disease, we have elected to refer to the cohort material as pAIDs.

The balance of the paediatric AI disease subjects' (SPA, PS, CEL and SLE) samples were accrued by our biorepository at the CHOP, which includes over 60,000 paediatric patients recruited and enrolled by the Center for Applied Genomics at CHOP. These individuals were ascertained for having a confirmed diagnosis of SPA, PS, CEL and SLE in the age range of 1–17 years during time of diagnosis and were required to fulfill clinical criteria for these respective disorders, as confirmed by a specialist. Only cases that upon EMR search were confirmed to have at least two or more in-person visits, at least one of which is with the specified ICD9 diagnosis code(s) were pursued for clinical confirmation (see Supplementary Table 12 for ICD-9 inclusion and exclusion codes). We used ICD9 codes previously identified and utilized for PheWAS or EMR-based GWAS^59^,^60^ and agreed upon by board-certified physicians.

Age- and gender-matched control subjects, including the EPI cohort of both generalized and focal idiopathic EPI (ICD-9 345.9 and 345.4, respectively), were identified from the CHOP-CAG biobank and ascertained by exclusion of any patient with any ICD-9 codes for disorders of autoimmunity or immunodeficiency^58^ (http://eicd9.com/). Research Ethics Boards at the CHOP and each of the collaborating centre, including: the Mount Sinai School of Medicine, University of Oxford, University of South Florida, the Children's Mercy Hospitals and Clinics, Texas Scottish Rite Hospital for Children, Murdoch Children's Research Institute, Oslo University Hospital, Cincinnati Children's Hospital Medical Center, McGill University, RCCS ‘Casa Sollievo della Sofferenza', University of Toronto, University of Edinburgh, Emory University, University of Naples ‘Federico II', Cedars Sinai Medical Center, Yorkhill Hospital for Sick Children, University of Miami Miller School of Medicine, Careggi University Hospital, University of Utah School of Medicine and Primary Children's Medical Center, approved this study.

Written informed consent was obtained from all subjects (or their legal guardians). Genomic DNA extraction and sample QC before and following genotyping were performed using standard methods^61^. To minimize confounding because of population stratification, we focused on only individuals of European ancestry, as determined by both self-reported ancestry and principle component analysis, PCA) in the present study (see below and Supplementary Fig. 4).

### Genotyping and QC

All samples were genotyped at the CAG on the HumanHap550 or 610 BeadChip arrays. Although some published analyses using GWAS data to derive heritability estimates have applied whole-genome imputation because of the presence of samples with non-matching platforms, this is not ideal given (i) added risk of artefacts and (ii) consequent variations in coverage (genotyping density) across the genome. Without adding significant additional information, this can result in biased heritability estimates unless careful corrections are made to apply additional down-sampling/weighting of more densely imputed regions^31^,^48^,^49^. As over 90% of the markers on the two arrays are shared, whole-genome imputation was not necessary and we utilized only the set of directly overlapping genotyped SNPs in the analysis (∼500,000).

After extracting the overlapping SNPs from the two platforms, SNPs with a low genotyping rate <95%, low MAF (<0.01) or significantly departing from the expected Hardy–Weinberg equilibrium (*P*<0.01) were excluded. Samples with low average genotyping call rate (<95%) or determined to be of outliers of European ancestry by PCA (Any of the top ten principal components (PCs) >6.0 standard deviations as reported by SMARTPCA/EIGENSTRAT^68^) were removed. In addition, one of each pair of distantly related individuals, as determined by Identify-by-State analysis (>0.05), was excluded, such that the largest sample size would be retained in the final cohort.

Web-based access to all novel data included in this manuscript is available through our website at http://www.caglab.org.

### Population stratification correction

The final cohort, following all above-noted QC, included a total of 4,956 pAID cases inclusive of 9 pAIDs and 27,451 population-matched controls, as well as a cohort consisting of 819 cases of paediatric-onset EPI. To avoid confounding, we assigned individuals fitting the diagnosis criteria for two or more pAIDs to the smaller disease cohort by sample size. No individual was included twice. To ensure that the markers tested across the cohorts were consistent, we included only SNPs that passed all QC criteria (461,301 SNPs). The filtered SNPs were tested in cases and controls for association with disease and used for the estimation of the genetic relationship matrix (see below). We used a logistic regression equation to estimate ORs/betas, 95% confidence intervals and *P*-values for trend, using additive coding for genotypes (0,1,2 minor alleles). We adjusted for gender and population stratification by including the binary gender and the first ten PCs (*GCTA)* from the PCA calculated from a set of 100,000 pruned SNPs as covariates in the logistic regression analyses^69^. From the results of the association testing, we determined the genomic inflation per disease-common control cohort. All disease-specific, case–control GWAS had *λ*_GC_ values at or below 1.04 with the exception of CD (1.09), consistent with that previously reported for this data set^29^. Final counts from each pAID cohort, included controls and genomic inflation calculated from median *χ*^2^ association test statistics are reported in Table 1.

### Estimation of the variance components for each pAID

Only individuals and SNPs that passed all QC metrics were used to estimate the variance components for the ten diseases (nine pAIDs and one non-pAID condition EPI). For disease-specific analysis, the common set of controls were used for each case–control analysis cohort, after excluding individuals who are relatives up to within the 5th degree. The genetic relationship between individuals was estimated using (i) all autosomal SNPs, (ii) all autosomal SNPs excluding the extended *MHC* (chr6:26.5–34 Mb) and (iii) SNPs only found on the *X-chromosome* (*ChrX*).

We applied the previously described linear mixed model method for estimating whole-genome SNP-based heritability using both common and low-frequency variants, which is implemented in the software *GCTA*. We estimated the genetic variance associated with genome-wide SNPs on the observed scale (SNP-heritability or SNP-*h*^*2*^)^70^, conditioning on the top 20 ancestry PCs derived from a pruned set of ∼100,000 independent SNPs across the same data set (that is, *PLINK* --indep-pairwise 50 10 0.2) obtained also using *GCTA*. As our phenotypes are dichotomous traits, we subsequently transformed these results to the liability scale based on approximately observed disease prevalence at our centre for each trait (Table 1). Note that the total control sample size utilized varied slightly as we optimized our analysis to maximize the retained sample size when conservatively removing distantly related individuals during QC. As we excluded rare variants (MAF<0.01), these variants are therefore not included in the heritability estimates attributable to genetic variation.

### Joint heritability across pAID pairwise combinations

We estimated the genetic correlation in disease risk for each of other pAID pairs using a bivariate linear mixed model, as described previously^17^. For each pairwise analysis, the pooled control samples passing QC were randomly allocated to the two diseases evenly and the top 20 PCs were again included as covariates. By jointly analysing a pair of cohorts, these results estimate both the SNP-*h*^*2*^ of liability to both diseases and an estimate of the SNP-genetic correlation between these liabilities. We determined the significance of the *rG* using a likelihood ratio test by fixing the genetic correlation at zero^17^. Significantly positive (or negative) *rG's* should reflect a shared (or disparate) genetic background, as a positive (negative) *rG* means that the correlation in the genetic variance components are higher (lower) between case subjects than between the case subjects and the respective control cohorts.

### Genome-wide pairwise sharing analysis

We applied a novel test to detect the presence of SNPs anywhere in the genome that are simultaneously associated with each of two diseases; these SNPs are the genetic risk factors shared by that pair of pAIDs. Most existing tests require choosing a significance threshold to determine which SNPs are associated with which disease, but it is unknown how best to choose this threshold. Our method is threshold-free and requires no tuning parameters. Specifically, for any two diseases, we converted the *P*-values for all SNPs in the genome into *Z*-scores, such that for example:


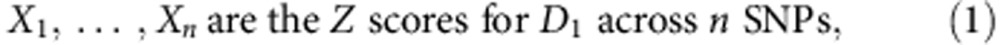



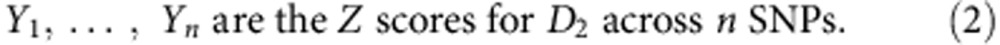


The test statistic, *γ*, used to detect genetic sharing between two diseases is


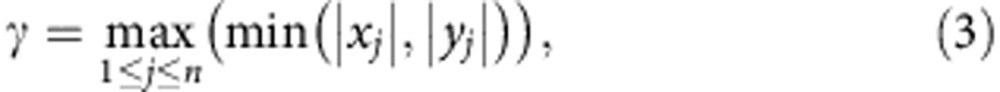


which is the maximum of the pairwise minima of the signals across all of the *n* SNPs. The rationale is that if SNP *j* is associated with both *D*_*1*_ and *D*_*2*_, the magnitudes of both *X*_*j*_ and *Y*_*j*_ should be large. The more shared SNPs there are, the greater the likelihood that the maximum of the pairwise minimal values will be large. Under the null hypothesis that any genetic sharing is due only to chance, *γ* should be relatively small. We can obtain the *P*-value of this statistic by permuting the labels of the *Z*-scores relative to each other in order to simulate the null hypothesis. In fact, these *P*-values can be calculated analytically using a hypergeometric distribution, and no actual permutation is needed. Note that no significance threshold is required. This test was performed for all 45 pairwise pAID combinations (hence, the reported *P*-values are Bonferroni-adjusted for 45 independent tests).

### Disease prediction using a linear SVM

Given that we observed relatively high rates of heritability across many of pAIDs, we sought to evaluate the utility of genome-wide SNP data in predicting pAID disease liability, using a previously described SVM pipeline that can be applied to GWAS results for a dichotomous trait^52^.

We identified SNPs to be used as predictors based on the strength of association with a given disease in a training set, testing graded *P*-value thresholds (*P*<1 × 10^−5^, 1 × 10^−6^, 1 × 10^−7^, 1 × 10^−8^, 1 × 10^−9^) for selecting SNP predictors, where the *P*-value is derived from the case–control association testing using samples in the training data set. We used each set of SNPs passing the tested threshold to then train the linear SVM model.

We then validated the SVM model by testing the accuracy of disease liability predictions for each of the nine pAIDs in the remaining independent sample set. We reported the prediction performance as the mean and maximum AUC achieved in both the training and validation sets (Fig. 3 and Supplementary Table 11).

## Additional information

**How to cite this article:** Li, Y. R. *et al*. Genetic sharing and heritability of paediatric age of onset autoimmune diseases. *Nat. Commun.* 6:8442 doi: 10.1038/ncomms9442 (2015).

## Supplementary Material

Supplementary Information Supplementary Figures 1-4, Supplementary Tables 1-12 and Supplementary References.

## Figures and Tables

**Table 1:** Summary of cohorts included.

**Disease**	**Full disease name**	**Cases**	**Controls**	GIF*	**Prevalence**†
CD	Crohn's disease	1,848	27,457	1.086	3.00E−03
CEL	Celiac disease	137	27,435	1.006	1.00E−02
CVID	Common variable immunodeficiency disorder	304	27,492	1.010	1.00E−04
EPI	Epilepsy	754	26,122	1.027	1.00E−04
JIA	Juvenile idiopathic arthritis	1,112	27,131	1.000	2.00E−03
PS	Psoriasis	85	27,474	1.012	1.00E−03
SLE	Systemic lupus erythematosus	252	27,525	1.019	1.00E−04
SPA	Spondyloarthropathy	98	27,483	1.020	1.00E−04
T1D	Type 1 diabetes	664	27,395	1.062	5.00E−03
UC	Ulcerative colitis	854	27,482	1.041	1.00E−03

GIF, genomic inflation factor; SNP, single-nucleotide polymorphism.

^*^GIF is provided for each cohort and included all SNPs (including *ChrX* and the extended *MHC*).

^†^Prevalence estimates used here are those made based on observations at our center.

**Table 2:** Contribution of autosomal, autosomal with extended MHC removed (exMHC) and ChrX variations to pAID heritability (*h* ^2^).

**Disease**	***h***^**2**^**(auto)**	**s.e.**	***P***	***h***^**2**^**(exMHC)**	**s.e.**	***P***	%MHC*	**ChrX**	**s.e.**	***P***
CD	0.454	0.025	<1.00E–04	0.447	0.025	<1.00E–04	1.54	0.014	0.004	2.35E–04
CEL	0.447	0.362	1.06E–01	0.337	0.361	1.74E–01	24.72	0.048	0.058	1.89E–01
CVID†	0.181	0.063	1.72E–03	0.167	0.063	3.66E–03	8.12	NA	NA	NA
EPI	0.168	0.027	1.05E–10	0.163	0.027	3.90E–10	2.91	0.010	0.005	1.21E–02
JIA	0.727	0.037	<1.00E–04	0.650	0.037	<1.00E–04	10.66	0.027	0.007	4.91E–06
PS	0.949	0.381	5.90E–03	0.949	0.380	5.87E–03	−0.02	0.003	0.061	4.82E–01
SLE	0.206	0.076	3.16E–03	0.202	0.076	3.74E–03	1.89	0.013	0.013	1.60E–01
SPA	0.370	0.192	2.45E–02	0.310	0.191	4.91E–02	16.17	−0.029	0.028	1.74E–01
T1D	0.863	0.070	<1.00E–04	0.581	0.069	<1.00E–04	32.66	0.028	0.012	5.27E–03
UC	0.386	0.041	<1.00E–04	0.363	0.041	<1.00E–04	5.84	0.012	0.007	3.38E–02

CD, crohn's disease; CEL, celiac disease; CVID, common variable immunodeficiency disorder; EPI, epilepsy; JIA, juvenile idiopathic arthritis; NA, not applicable; pAID, paediatric autoimmune disease; PS, psoriasis; SLE, systemic lupus erythematosus; SPA, spondyloarthropathy; T1D, type 1 diabetes; UC, ulcerative colitis.

P-values (*P)* are based on results from the restricted maximum likelihood estimate (likelihood ratio test). Error bars represent standard error.

^*^Percentage contribution of the extended *MHC* to total autosomal SNP-*h*^*2*^

^†^REML estimates could not be made due to limited common SNP variability among this cohort on the *X-chromosome*

**Table 3:** pAID joint heritabilities or genetic correlation (rG) reaching nominal significance.

**pAID pair**	**rG (auto)**	**s.e.**	***P*****val**	**rG (exMHC)**	**s.e.**	***P*****_nominal**	***P*****_adj**
CVID-JIA	0.343	0.127	1.22E–03	0.354	0.142	2.47E–03	2.23E–02
EPI-JIA	−0.150	0.079	2.95E–02	−0.142	0.085	4.87E–02	0.44
EPI-UC	0.197	0.103	2.77E–02	0.248	0.108	1.06E–02	0.10
PS-T1D	−0.241	0.139	3.29E–02	−0.282	0.167	3.74E–02	0.34
PS-UC	−0.316	0.169	2.31E–02	−0.289	0.171	3.76E–02	0.34
SLE-CD	−0.266	0.120	8.25E–03	−0.255	0.121	1.15E–02	0.10
SPA-CD	−0.215	0.138	4.64E–02	−0.235	0.156	4.67E–02	0.42
T1D-CD	0.096	0.053	3.45E–02	0.142	0.064	1.33E–02	0.12
UC-CD	0.659	0.069	<1.00E–04	0.674	0.072	<1.00E–04	9.00E–04

Auto, autosomal; CD, crohn's disease; CEL, celiac disease; CVID, common variable immunodeficiency disorder; EPI, epilepsy; JIA, juvenile idiopathic arthritis; NA, not applicable; pAID, paediatric autoimmune disease; PS, psoriasis; exMHC, MHC excluded; SLE, systemic lupus erythematosus; SPA, spondyloarthropathy; T1D, type 1 diabetes; UC, ulcerative colitis.

*P*-values *(P)* are based on results from the restricted maximum likelihood estimate (likelihood ratio test). *P*_adj is made using a Bonferonni-adjustment for nine pairwise tests for each disease.
