# Supplementary material for: Genetic sharing and heritability of paediatric age of onset autoimmune diseases
Source: Nat Commun. 2015 Oct 9;6:8442. doi: 10.1038/ncomms9442 (PMC4633631; doi:10.1038/ncomms9442)
Supplement: Supplementary Information — Supplementary Figures 1-4, Supplementary Tables 1-12 and Supplementary References. [file ncomms9442-s1.pdf]

Supplementary Figure 1. SNP-heritability calculations reported by prior studies for A. Relevant AI diseases and B. All available diseases curated.

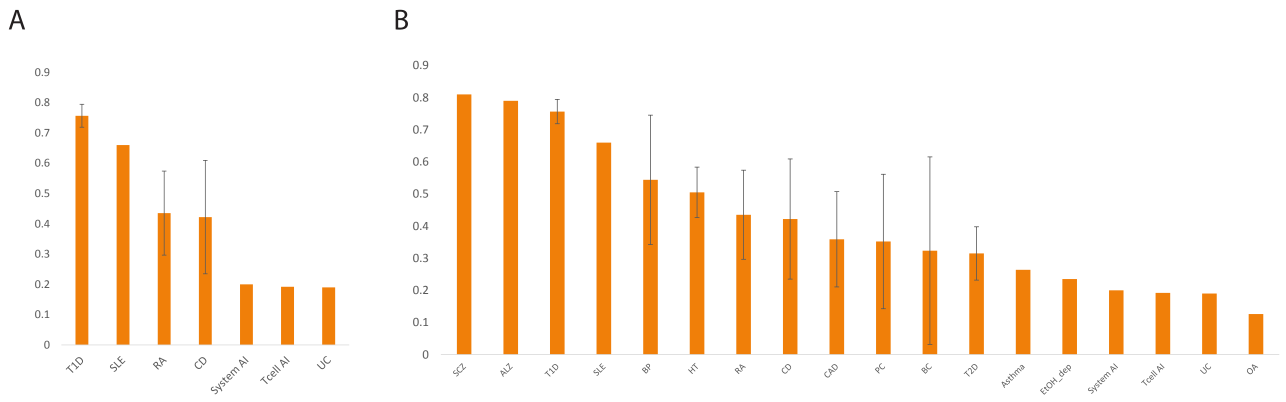

Raw data used from prior GWAS SNP-heritability estimates are provided in **Supplementary Table 1C.**

Supplementary Figure 2 Partitioning phenotypic variance to genetic and non-genetic components for all ten traits examined.

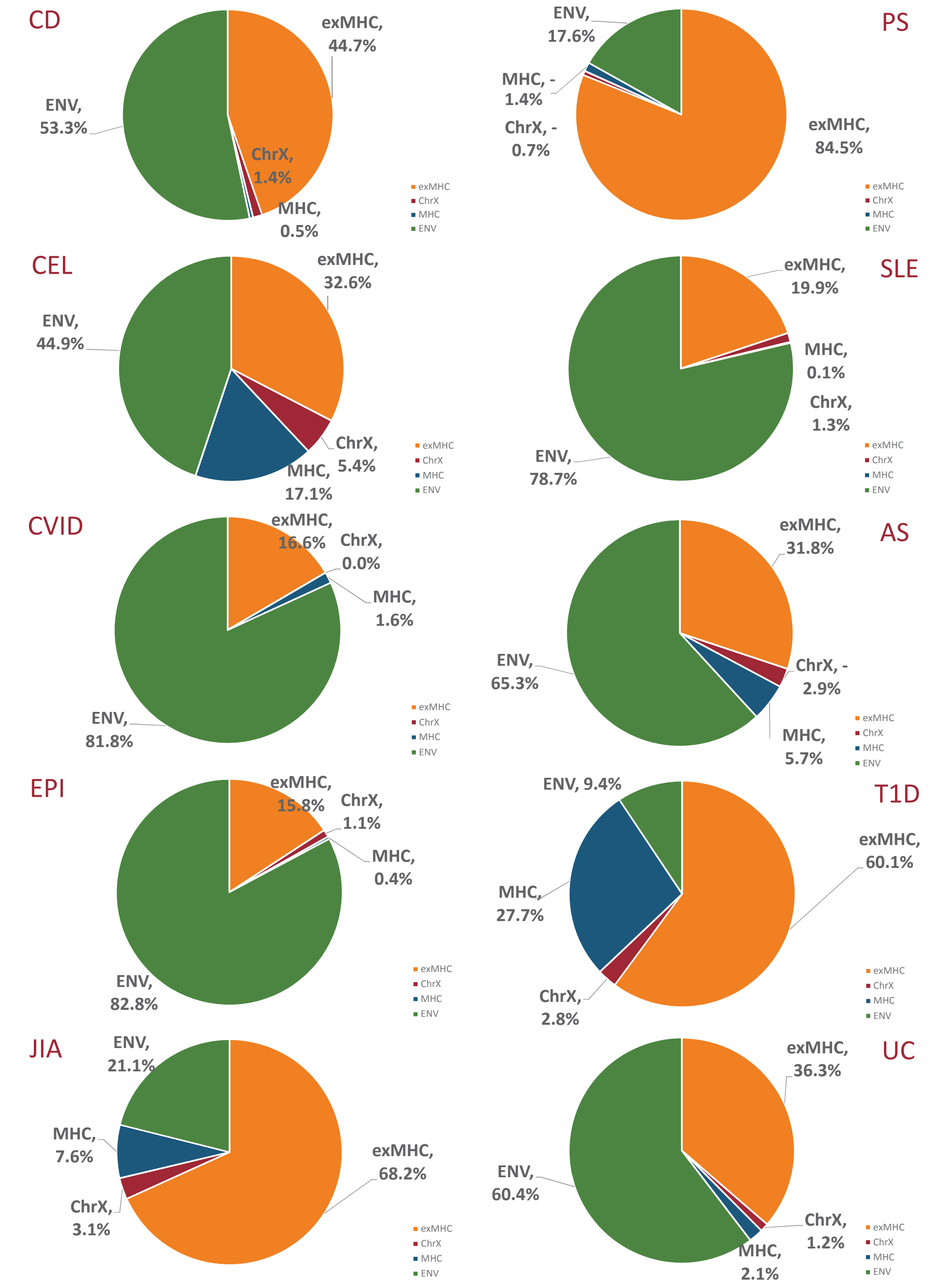

Pie chart includes Non-genetic *(ENV, Green)* and Genetic components, which is sub-divided into contributions from the autosomal regions excluding the extended *MHC* (ex*MHC*, Orange), the extended *MHC* (*MHC*, Blue), and the *X-chromosome* (*ChrX*, Red).

Supplementary Figure 3 Effect of LD-pruning on SNP-heritability estimates.

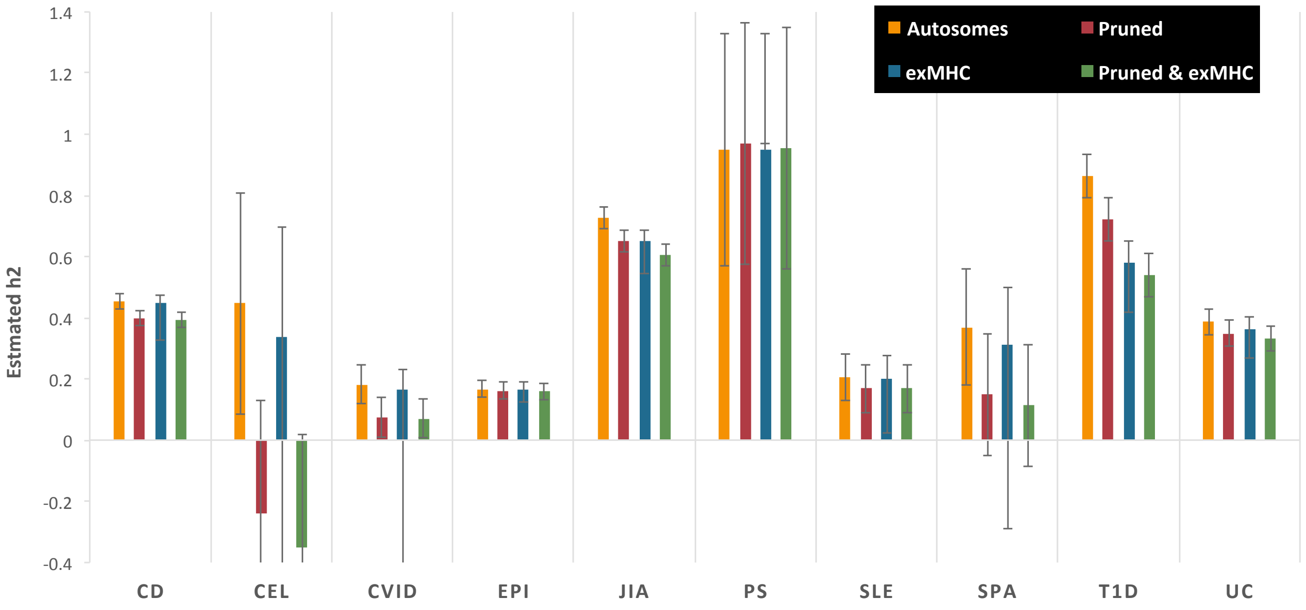

Autosomal SNP-h^2 estimations using all genotyped markers including the extended *MHC* (Orange) is significantly greater than when the markers are LD-pruned (Red). When the *MHC* is removed, the difference in SNP-h^2 calculated between using the full autosomal genotyped SNPs (Blue) as compared those that are LD-pruned (Green) is minimal.

Supplementary Figure 4 Genetically-inferred ancestry estimates of included cohorts based on principal components analysis (PCA)

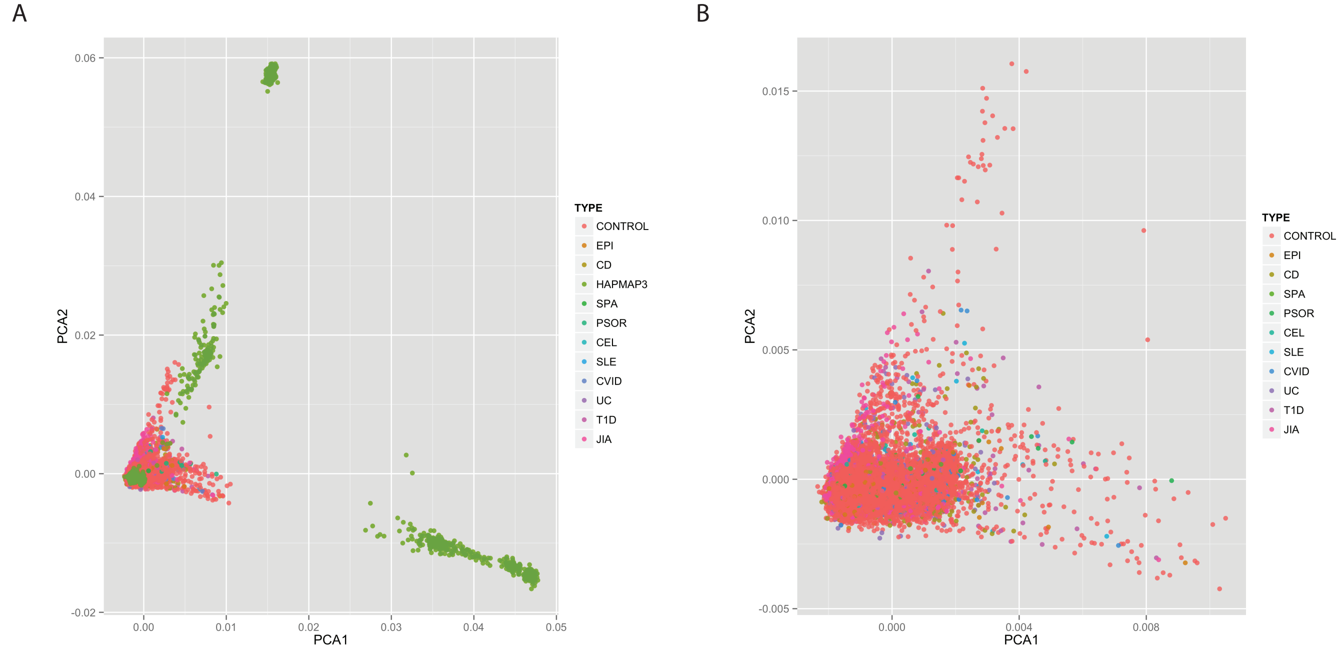

Top two principal components from PCA of genome wide SNP genotypes in the genotyped study cohort with (A) and without (B) the HapMap 3 reference panel dataset subjects. Plots are color-coded by the pAID cohorts either with or without the HapMap subjects. Populations and cohorts sizes are as specified in **Table 1A.**

Supplementary Table 1 Reported pAID prevalence in the Western Hemisphere in Caucasoid populations

pAID	Data 1	REF	Data 2	REF	Data 3	REF	Data 4	REF
SPA	687/217,000	1	62/10000	2	52/10000	3		
PS	10788/5,472,032	4	114,521/7,533,475	5	548/21,921	6	33981/1359240	7
CEL	2722/5,472,032	4	6/813	8	4/2230	9	10/1002	10
SLE	1732/5,472,032	4	62/2,210,389	11	51/100,000	12		
CVID	87500/297,386,000	13	52.5/1,000,000	14	1/30,000	15		
UC	20669/5,472,032	4	15,873/8,997,731	16	8/100,000	17		
T1D	51783/5,472,032	4	980/11,194,478	18	4808/1,885,451	19		
JIA	87/445856	20	294,000/295,753,000	21	78/364,939	22		
CD	12309/5,472,032	4	13,918/8,997,731	16	140/496,280	23		

Supplementary Table 2 Reported pAID heritability estimates in the Western Hemisphere in Caucasoid populations

pAID	Data 1	Ref 1*	Data 2	Ref 2*	Data 3	Ref 3*
CD	58%	24	80%	25		
CEL	59%	26	87%	27	0.57	28
JIA	65%	29	53%	30	0.6	30
PS	68%	31	91%	32		
SLE	66%	33	66%	34		
SPA	90%	35	90%	36		
T1D	88%	37	88%	38		
UC	42%	24	53%	25		

Supplementary Table 3 Published SNP-h^2 estimates from literature.

Trait	*So*[39]	*Zaitlen*[40]	*Yang*[41]	*Chen*[42]	*Lee*[43]	*Speed*[44]	*Speed*[45]
Method	Variants	REML	REML	GREML	GREML	GREML	LDAK
Scale	Liability	Liability	Liability	Liability	Liability	Observed	Observed
ALZ	0.79	--	--	--	--	--	--
BP	0.77	--	0.32	--	0.35	0.69	0.59
BC	0.53	0.117	--	--	--	--	--
CAD	0.49	0.146	--	--	--	0.39	0.41
CD	0.55	--	0.18	0.26	--	0.54	0.58
PC	0.5	0.204	--	--	--	--	--
SCZ	0.81	--	--	--	--	--	--
SLE	0.66	--	--	--	--	--	--
T1D	0.8	--	0.25 (exMHC)	--	--	0.73	0.74
T2D	0.42	0.254	--	--	0.21	0.35	0.34
AI (T-cell)*	--	0.192	--	--	--	--	--
AI (Systemic)*	--	0.2	--	--	--	--	--
Asthma	--	0.264	--	--	--	--	--
RA	--	0.261	--	--	0.39	0.57	0.52
OA	--	0.126	--	--	--	--	--
Alcohol Dependence	--	0.235	--	--	--	--	--
UC	--	--	--	0.19	--	--	--
HT	--	--	--	--	0.575	0.42	0.52

See text for details and discussion. Disease or trait is listed on left and each column heading corresponding to first author last name of a reference as cited by superscript. *Per *Zaitlen* et al, AI disease that the authors classified as being 'T-cell mediated' or 'Systemic' (ie. RA/SLE/SSc/AS). References for each citation are as annotated in superscripts following each first author's last name.

Supplementary Table 4 Percentages of total phenotypic variance explained by genetic variance (the autosome excluding the *MHC*, the *MHC* and the X-chromosome) and non-genetic variance (ENV).

pAID	*Auto exMHC*	*ChrX*	*MHC*	ENV
**CD**	44.7%	1.4%	0.5%	53.3%
**CEL**	32.6%	5.4%	17.1%	44.9%
**CVID**	16.6%	0.0%	1.6%	81.8%
**EPI**	15.8%	1.1%	0.4%	82.8%
**JIA**	68.2%	3.1%	7.6%	21.1%
**PS**	84.5%	-0.7%	-1.4%	17.6%
**SLE**	19.9%	1.3%	0.1%	78.7%
**AS**	31.8%	-2.9%	5.7%	65.3%
**T1D**	60.1%	2.8%	27.7%	9.4%
**UC**	36.3%	1.2%	2.1%	60.4%

Supplementary Table 5 Lead GWAS association signals across the extended *MHC* for each pAID

pAID	Marker	POS (hg19)	MAF	A2	P	BETA	SE
CD	rs1626392	29840223	0.21	G	2.3E-07	-0.22	0.04
CEL	SNP_DQB1_32740731	32740731	0.22	C	1.0E-26	-1.60	0.15
CVID	SNP_B_31432003_C	31432003	0.19	A	3.8E-08	0.61	0.11
JIA	AA_DRB1_181_32657335_x	32657335	0.03	A	1.6E-99	-2.57	0.12
PSOR	HLA_B_1302	31431272	0.03	A	3.1E-04	-1.74	0.48
SLE	rs537160	32024379	0.30	C	4.5E-08	-0.55	0.10
SPA	HLA_B_2705	31431272	0.03	A	1.9E-11	-2.88	0.43
T1D	AA_DQB1_57_32740666_A	32740666	0.34	A	3.0E-170	-1.72	0.06
UC	SNP_DRB1_32660090	32660090	0.31	A	2.5E-15	0.43	0.05

* P (Presence) or A (Absence) of the indicated HLA 4-digit allele, AA (amino acid [ie. lysine (K) or alanine (A)]), deletion (x). Details of the notion for the markers are as described by *Jia et al*.[46] A2: Alternative allele or marker. For deletions, A denotes 'Absence of'. P: P-value. MAF: Minor Allele Frequency. POS: position in the hg19 human genome reference. BETA: effect size on logistic regression. SE: standard error.

Supplementary Table 6 Population-based estimates of heritability (POP-h^2) attributable to the *MHC* from literature

pAID	Total h^2 (1)	Total h^2 (2)	Mean h^2	% MHC	REF
SPA	0.90	0.90	0.90	0.25	3
PS	0.68	0.91	0.80	0.50	47
CEL	0.59	0.87	0.73	0.40	48,49
SLE	0.66	0.66	0.66	0.10	50
UC	0.42	0.53	0.48	0.60	51
T1D	0.40	0.62	0.88	0.50	51
JIA	0.65	0.53	0.59	0.33	51
CD	0.58	0.80	0.69	0.10	51

See Supplementary Table 1B for details of the total heritability references. REF refers to references of the percentage heritability attributable to the *MHC.*

Supplementary Table 7 Estimates of SNP-h^2 attributable to the *MHC* using other GWAS datasets

Disease	SNP-h^2	SNP-h^2 *MHC*	% *MHC*
BPD	0.98	0	0.00%
CAD	0.41	0	0.00%
CD	1	0.02	2.00%
HT	0.48	0	0.00%
RA	0.57	0.13	22.81%
T1D	0.65	0.44	67.69%
T2D	0.52	0	0.00%

Supplementary Table 8 Known HLA haplotypes associated with AI Diseases

Disease	HLA Associated	REF
Ankylosing spondylitis	Class I:<br>- B27<br>Class II:	52
Asthma	Class I:<br>Class II:<br>- DRB1*03 haplotype<br>Class III:<br>- TNFA-308	53
Autoimmune thyroid diseases (Grave's & Hashimoto)	Class I:<br>Class II:<br>- DR3 (DRB1*03; DRB1*Arg74),<br>- DR4 (in Hashimoto's thyroiditis)	52,54
Celiac disease	Class I:<br>Class II:<br>- DQ2 (DRB1*0301-DQA1*0501-DQB1*0201)<br>- DQ8 (DRB1*04-DQA1*0301-DQB1*0302)	55,56
Crohn's disease	Class I:<br>Class II:<br>- DRB1*0103-DQB1*0501<br>- DRB1*04	51,57
Multiple sclerosis	Class I:<br>- A*02:01<br>Class II:<br>- DR2 (DRB1*1501, DQA1*0102, DQB1*0602)<br>- DRBI* 0501<br>- DRB1*03:01, *13:03<br>- DQB1*02:01<br>- DPB1*03:01	58,59
Psoriasis	Class I:<br>- HLA-B*57<br>- HLA-B*40 (Decreased susceptibility)<br>- Cw*0602<br>- Cw*1203<br>Class II:<br>Class III:<br>- HCP5	60

Rheumatoid arthritis	Class I:<br><br>- HLB*08<br>Class II:<br><br>- DRBI *0401, *0404, *0101, *0405, *0101, *1001, *0901<br>Class III:<br><br>- TNF	61,62
Systemic lupus erythematosus	Class I:<br><br>Class II:<br><br>- DR3 (DRB1*0301-DQB1*0201),<br>- DR2 (DRB1*1501-DQB1*0602),<br>- DR8 (DRB1*0801-DQB1*0402)<br>- DRBI*0301<br>Class III:<br><br>- SCIV2L/ CFB/ RDBP/ DOM3Z/STK19<br>- C4A, C4B	63
Type 1 diabetes	Class I:<br><br>- HLA-A<br>- HLA-B. Protective effect – DQB1*0602<br>Class II:<br><br>- DQ2 (DRB1*0301-DQA1*0501-DQB1*0201)<br>- DQ8 (DRB1*04-DQA1*0301-DQB1*0302)	64
Ulcerative colitis	Class I:<br><br>Class II:<br><br>- DRB1*0103-DQB1*0501<br>- DRB1*1502-BTNL2)<br>- DRB1* 0501	51,65,66
Multiple Autoimmune diseases	Class I:<br><br>- HLA-A1<br>- HLA-B8<br>Class II:<br><br>- HLA-DR3	51
Sarcoidosis	Class I:<br><br>Class II:<br><br>- HLA-DRB1*03	67

Supplementary Table 9 Prevalence of AI Disease comorbidities among Western Caucasoid populations

Prevalence	Data	REF	Data	REF	Data 3	REF	Data4	REF
CEL-CD	--	--	4/356	8	--	--	--	--
CEL-CVID	--	--	--	--	--	--	--	--
CEL-JIA	0/408	4	--	--	--	--	--	--
CEL-SLE	--	--	4/356	8	--	--	--	--
CEL-T1D	12/408	4	8/356	8	96/9243	10	921/28958	5
CEL-PS	--	--	46/356	8	--	--	401/28958	5
CEL-SPA	--	--	2/356	8	--	--	--	--
CEL-UC	--	--	3/356	8	--	--	--	--
CVID-CD	1/52	68	--	--	--	--	--	--
CVID-JIA	4/52	68	--	--	--	--	--	--
CVID-SLE	1/116	69	--	--	--	--	--	--
JIA-T1D	4/443	20	0/53	70	--	--	--	--
JIA-CEL	10/151	71	1/53	70	--	--	--	--
PS-CD	95/10788	72	116/ 1353	73	72/1402	73	64/12502	74
PS-CEL	--	--	--	--	--	--	36/12502	75
PS-SLE	19/10788	72	--	--	--	--	--	--
PS-T1D	442/10788	72	--	--	--	--	--	--
PS-SPA	--	--	--	--	--	--	--	--
PS-UC	122/10788	72	166/1353	73	74/1402	73	60/ 12502	74
SLE-CD	13/1732	72	--	--	--	--	--	--
SLE-PS	19/1732	72	--	--	--	--	--	--
SLE-CEL	3/1732	72	--	--	--	--	--	--
SLE-T1D	46/1732	72	--	--	--	--	--	--
SLE-UC	25/1732	72	--	--	--	--	--	--
T1D-PS	442/51783	72	--	--	--	--	--	--
T1D-SLE	46/51783	72	--	--	--	--	--	--
T1D-CEL	108/51783	72	9/52	76	917/ 41566	77	33/ 1000	19
T1D-CEL	22/ 492	78	9/ 281	79	7/509	18		
T1D-UC	435/51783	72	--	--	--	--	--	--
T1D-CD	217/51783	72	--	--	--	--	--	--
T1D-JIA	4/443	16	--	--	--	--	--	--
UC-CD	--	--	--	--	2676/20699	72	--	--
UC-T1D	84/12185	80	63/7525	81	435/20669	72	--	--
UC-SPA	117/12185	--	9/976	23	7/84	82	7/273	83
UC-SLE	10/12185	--	18/7525	81	25/20669	72	--	--
UC-CEL	--	--	--	--	77/20669	72	--	--
UC-PS	424/12185	--	138/7525	81	122/20669	72	--	--
CD-UC	--	--	--	--	2676/12309	72	--	--
CD-CEL	--	--	--	--	101/12309	72	--	--
CD-T1D	35/7988	80	43/4021	81	217/12309	72	--	--
CD-SLE	12/7988	80	8/4021	81	13/12309	72	--	--
CD-PS	317/7988	80	75/4021	81	95/12309	72	--	--
CD-SPA	107/7988	80	6/483	23	9/78	82	8/133	83

Supplementary Table 10 Full results from the bivariate co-heritability analysis showing genetic correlation estimates for all 45 pairwise AI disease combinations

	Auto *rG*	Auto *SE*	Auto *P*	exMHC *rG*	exMHC *SE*	exMHC *P*	chrX *rG*	chrX *SE*	chrX *P*
CEL-CD	0.192	0.162	1.00E-01	0.211	0.188	1.05E-01	-0.17	0.31	0.29
CEL-CVID	0.199	0.391	2.99E-01	0.245	0.475	2.94E-01	NA	NA	NA
CEL-JIA	0.013	0.182	4.71E-01	0.121	0.239	2.96E-01	0.44	0.19	0.01
CEL-SLE	0.008	0.415	4.92E-01	0.054	0.498	4.56E-01	0.02	0.15	0.45
CEL-T1D	0.072	0.200	3.57E-01	-0.084	0.285	3.82E-01	-0.03	0.31	0.46
CEL-UC	-0.018	0.259	4.73E-01	-0.051	0.323	4.38E-01	0.11	0.31	0.37
CVID-CD	0.101	0.109	1.75E-01	0.115	0.116	1.56E-01	-1.00	124.42	0.50
CVID-JIA	0.343	0.127	1.22E-03	0.354	0.142	2.47E-03	-1.00	3.78	0.50
CVID-T1D	0.177	0.130	7.89E-02	0.207	0.167	9.92E-02	-1.00	291.00	0.50
CVID-UC	0.138	0.158	1.89E-01	0.134	0.170	2.12E-01	-1.00	34.60	0.50
EPI-CD	0.023	0.075	3.79E-01	0.003	0.077	4.85E-01	0.89	0.39	0.00
EPI-CEL	-0.099	0.264	3.54E-01	-0.078	0.304	3.99E-01	-0.17	0.25	0.26
EPI-CVID	-0.122	0.183	2.52E-01	-0.095	0.196	3.14E-01	-1.00	219.03	0.50
EPI-JIA	-0.150	0.079	2.95E-02	-0.142	0.085	4.87E-02	-0.04	0.19	0.42
EPI-PSOR	0.087	0.188	3.23E-01	0.030	0.189	4.38E-01	-0.36	0.35	0.15
EPI-SLE	0.153	0.176	1.92E-01	0.187	0.183	1.52E-01	-0.21	0.15	0.10
EPI-SPA	0.090	0.215	3.37E-01	0.073	0.240	3.80E-01	0.05	2.15	0.49
EPI-T1D	-0.017	0.086	4.20E-01	0.022	0.105	4.18E-01	0.09	0.31	0.39
EPI-UC	0.197	0.103	2.77E-02	0.248	0.108	1.06E-02	-0.63	0.29	0.01
JIA-CD	0.019	0.049	3.50E-01	0.030	0.053	2.85E-01	0.02	0.21	0.47
PSOR-CD	-0.089	0.113	2.15E-01	-0.100	0.115	1.92E-01	0.31	0.45	0.25
PSOR-CEL	-0.092	0.428	4.14E-01	-0.058	0.495	4.53E-01	0.58	0.36	0.05
PSOR-CVID	0.271	0.269	1.50E-01	0.284	0.282	1.49E-01	NA	NA	NA
PSOR-JIA	0.006	0.123	4.81E-01	0.042	0.131	3.77E-01	0.03	0.22	0.44
PSOR-SLE	-0.115	0.260	3.29E-01	-0.065	0.262	4.02E-01	0.32	0.20	0.07
PSOR-T1D	-0.241	0.139	3.29E-02	-0.282	0.167	3.74E-02	-0.27	0.39	0.25
PSOR-UC	-0.316	0.169	2.31E-02	-0.289	0.171	3.76E-02	-0.15	0.39	0.36
SLE-CD	-0.266	0.120	8.25E-03	-0.255	0.121	1.15E-02	0.07	0.19	0.37
SLE-CVID	-0.149	0.261	2.82E-01	-0.187	0.280	2.49E-01	1.00	6.04	0.50
SLE-JIA	0.115	0.115	1.58E-01	0.091	0.123	2.28E-01	-0.19	0.11	0.05
SLE-T1D	-0.011	0.126	4.65E-01	-0.021	0.153	4.46E-01	-0.10	0.22	0.34
SLE-UC	-0.010	0.151	4.74E-01	-0.020	0.157	4.50E-01	-0.29	0.19	0.07
SPA-CD	-0.215	0.138	4.64E-02	-0.235	0.156	4.67E-02	1.00	5.50	0.36
SPA-CEL	0.284	0.520	2.84E-01	0.383	0.700	2.76E-01	0.77	3.18	0.26
SPA-CVID	-0.079	0.320	4.02E-01	-0.083	0.363	4.09E-01	NA	NA	NA
SPA-JIA	0.116	0.138	1.95E-01	0.091	0.158	2.80E-01	-1.00	0.66	0.00
SPA-PSOR	0.171	0.322	2.94E-01	0.214	0.357	2.69E-01	1.00	2.96	0.22
SPA-SLE	0.101	0.302	3.68E-01	0.096	0.334	3.86E-01	0.80	2.41	0.13
SPA-T1D	0.158	0.164	1.58E-01	0.229	0.223	1.37E-01	-1.00	1.72	0.16
SPA-UC	-0.012	0.181	4.73E-01	-0.049	0.204	4.05E-01	-1.00	2.99	0.19
T1D-CD	0.096	0.053	3.45E-02	0.142	0.064	1.33E-02	0.22	0.34	0.26
T1D-JIA	0.064	0.055	1.26E-01	0.112	0.070	5.59E-02	-0.01	0.20	0.48
UC-CD	0.659	0.069	0.00E+00	0.674	0.072	0.00E+00	0.29	0.33	0.19
UC-JIA	0.046	0.067	2.49E-01	0.024	0.073	3.72E-01	0.30	0.21	0.08
UC-T1D	-0.090	0.070	9.79E-02	-0.095	0.086	1.33E-01	-0.14	0.32	0.33

Supplementary Table 11 Full AUC results from pAID liability prediction analysis

Trait	Training $P_{threshold}$	training			*VALIDATION*			
		min	avg	max	*MIN*	*AVG*	*MAX*	*STDEV*
JIA	1.00E-08	91.18%	91.50%	92.05%	*86.86%*	*89.27%*	*92.46%*	*2.06%*
JIA	1.00E-07	91.78%	92.35%	92.72%	*85.80%*	*88.65%*	*91.86%*	*2.21%*
JIA	1.00E-06	92.80%	93.16%	93.53%	*84.15%*	*88.09%*	*91.67%*	*2.58%*
JIA	1.00E-05	93.61%	93.88%	94.33%	*82.76%*	*87.06%*	*91.14%*	*2.56%*
JIA	1.00E-04	93.85%	94.29%	94.78%	*82.16%*	*86.13%*	*89.72%*	*2.62%*
JIA	1.00E-03	94.61%	95.07%	95.41%	*80.03%*	*84.80%*	*87.24%*	*2.60%*
CD	1.00E-08	69.65%	70.18%	71.34%	*64.69%*	*68.13%*	*70.69%*	*1.46%*
CD	1.00E-07	71.20%	72.07%	73.10%	*66.77%*	*69.42%*	*72.37%*	*1.59%*
CD	1.00E-06	74.94%	75.42%	76.29%	*67.90%*	*70.18%*	*73.69%*	*2.01%*
CD	1.00E-05	79.35%	80.02%	81.09%	*69.27%*	*71.84%*	*75.81%*	*1.95%*
CD	1.00E-04	84.36%	85.15%	85.87%	*70.28%*	*73.79%*	*76.69%*	*2.16%*
CD	1.00E-03	90.04%	90.61%	91.17%	*68.20%*	*70.96%*	*75.23%*	*2.28%*
UC	1.00E-08	61.55%	63.25%	64.78%	*56.96%*	*60.88%*	*64.77%*	*2.44%*
UC	1.00E-07	64.72%	66.23%	67.53%	*58.71%*	*62.51%*	*67.24%*	*2.47%*
UC	1.00E-06	70.24%	71.46%	72.66%	*59.27%*	*63.10%*	*67.73%*	*2.54%*
UC	1.00E-05	77.77%	79.42%	80.19%	*60.29%*	*64.99%*	*68.58%*	*2.89%*
UC	1.00E-04	86.38%	87.01%	87.64%	*62.96%*	*66.01%*	*69.39%*	*2.07%*
UC	1.00E-03	92.93%	93.59%	94.23%	*59.81%*	*64.45%*	*68.26%*	*2.87%*
T1D	1.00E-08	86.95%	87.28%	87.58%	*84.79%*	*87.29%*	*91.32%*	*2.12%*
T1D	1.00E-07	87.27%	87.59%	87.90%	*84.93%*	*87.48%*	*91.31%*	*1.98%*
T1D	1.00E-06	88.17%	88.34%	88.60%	*85.43%*	*87.54%*	*90.96%*	*1.82%*
T1D	1.00E-05	89.39%	89.90%	90.57%	*85.82%*	*87.37%*	*91.16%*	*1.81%*
T1D	1.00E-04	93.14%	93.65%	94.49%	*84.15%*	*85.74%*	*88.84%*	*1.55%*
T1D	1.00E-03	97.90%	98.01%	98.14%	*79.65%*	*82.02%*	*85.61%*	*1.63%*
CEL	1.00E-08	69.07%	70.83%	72.37%	*56.95%*	*71.25%*	*84.73%*	*7.57%*
CEL	1.00E-07	68.89%	71.02%	72.28%	*55.74%*	*70.62%*	*83.88%*	*7.70%*
CEL	1.00E-06	70.31%	71.81%	74.98%	*53.79%*	*69.74%*	*84.75%*	*8.39%*
CEL	1.00E-05	73.73%	75.61%	77.76%	*51.54%*	*67.50%*	*80.26%*	*8.06%*
CEL	1.00E-04	89.07%	90.65%	92.89%	*49.69%*	*63.37%*	*74.15%*	*7.75%*
CEL	1.00E-03	99.28%	99.50%	99.75%	*41.18%*	*56.87%*	*68.21%*	*8.07%*
PS	1.00E-08	54.09%	54.09%	54.09%	*42.67%*	*42.67%*	*42.67%*	*0.00%*
PS	1.00E-07	54.09%	57.32%	60.54%	*39.25%*	*40.96%*	*42.67%*	*2.42%*
PS	1.00E-06	54.56%	60.02%	67.04%	*33.00%*	*41.71%*	*54.52%*	*7.29%*
PS	1.00E-05	69.84%	78.70%	83.34%	*27.03%*	*38.49%*	*60.79%*	*9.19%*
PS	1.00E-04	94.43%	96.28%	97.30%	*31.41%*	*46.69%*	*60.78%*	*8.19%*
PS	1.00E-03	99.97%	99.98%	99.99%	*45.38%*	*51.31%*	*57.79%*	*3.39%*
SLE	1.00E-08	60.11%	61.39%	63.13%	*46.10%*	*46.98%*	*48.12%*	*0.84%*
SLE	1.00E-07	59.21%	62.31%	64.03%	*47.85%*	*52.39%*	*58.92%*	*3.68%*
SLE	1.00E-06	62.23%	64.80%	67.67%	*50.69%*	*57.02%*	*69.71%*	*5.42%*
SLE	1.00E-05	67.89%	71.98%	78.69%	*45.44%*	*57.40%*	*68.04%*	*6.31%*
SLE	1.00E-04	87.24%	89.77%	91.69%	*51.67%*	*56.40%*	*61.95%*	*3.46%*

SLE	1.00E-03	98.99%	99.19%	99.66%	*51.49%*	*59.05%*	*70.54%*	*7.27%*
AS	1.00E-07	49.88%	55.32%	63.57%	*47.22%*	*54.53%*	*60.33%*	*4.44%*
AS	1.00E-06	57.16%	64.71%	68.91%	*47.99%*	*57.76%*	*67.43%*	*6.05%*
AS	1.00E-05	75.69%	78.57%	80.91%	*51.72%*	*59.99%*	*68.12%*	*4.86%*
AS	1.00E-04	94.32%	95.59%	96.75%	*45.23%*	*49.94%*	*59.43%*	*5.36%*
AS	1.00E-03	99.95%	99.99%	100.00%	*44.34%*	*53.28%*	*61.24%*	*5.46%*
THY	1.00E-08	62.62%	62.62%	62.62%	*48.14%*	*48.14%*	*48.14%*	*0.00%*
THY	1.00E-07	57.66%	62.00%	67.77%	*44.66%*	*48.91%*	*56.98%*	*4.77%*
THY	1.00E-06	59.48%	69.51%	75.99%	*42.32%*	*52.23%*	*61.36%*	*6.36%*
THY	1.00E-05	77.23%	82.02%	89.03%	*30.03%*	*50.14%*	*67.09%*	*11.91%*
THY	1.00E-04	96.49%	97.10%	97.96%	*35.56%*	*53.57%*	*67.67%*	*12.85%*
THY	1.00E-03	99.97%	99.99%	100.00%	*53.11%*	*60.88%*	*73.24%*	*6.48%*
CVID	1.00E-08	60.23%	61.57%	62.64%	*52.47%*	*60.65%*	*68.12%*	*4.14%*
CVID	1.00E-07	61.58%	62.77%	63.72%	*53.01%*	*60.43%*	*64.14%*	*3.79%*
CVID	1.00E-06	63.67%	68.46%	71.38%	*56.18%*	*61.82%*	*68.87%*	*4.37%*
CVID	1.00E-05	73.73%	77.66%	82.24%	*58.44%*	*63.57%*	*67.32%*	*3.39%*
CVID	1.00E-04	88.53%	91.41%	92.73%	*57.78%*	*64.18%*	*72.69%*	*4.57%*
CVID	1.00E-03	98.80%	99.11%	99.27%	*50.68%*	*59.55%*	*64.83%*	*5.04%*

Supplementary Table 12 ICD9 'diagnosis' search terms used to select subjects for inclusion from EMR datasets

pAID	ICD9 Search Terms and Codes
THY	%Chronic%Thyroiditis% | %Grave% | %Hashimoto% | 242.0% | 245.0% | 245.2%
SPA	%Ankyl%Spond%litis% | %Spondyloarthropathy% | 720% | 720%
PSOR	%Psoriasis% | 696.10%
CEL	%Celiac% | 579.00%
SLE	%Systemic%Lupus%Erythematosus% | 710.0%
CVID	%Variable%Immunodefi% | 279.06%
UC	%Ulcerative%Colitis% | 556%
T1D	%Type%1%Diabetes% | 250._1% | 250._3%
JIA	%Enthe%rthritis% | %Idiop%rthritis% | %Juvenile%rthritis% | %Mono%rthritis% | %Oligo%rthritis% | %Poly%rthritis% | %Psor%rthritis% | %Rheum%rthritis% | %System%Arthritis% | 714% | 716.2% | 716.5% | 716.6% | 716.8% | 716.9%
CD	%Crohn% | 555%

*NOTE: ICD9 codes used for EPIC-SQL case identification by patient diagnosis [% = wildcard (0 or more characters), _ = wildcard (exactly 1 character)]. *EMR: Electronic Medical Records*

Supplementary References

1. Gran, J. T., Husby, G. & Hordvik, M. Prevalence of ankylosing spondylitis in males and females in a young middle-aged population of Tromsø, northern Norway. *Ann. Rheum. Dis.* **44,** 359–367 (1985).

2. Saraux, A. *et al.* Prevalence of rheumatoid arthritis and spondyloarthropathy in Brittany, France. *J. Rheumatol.* **26,** 2622–2627 (1999).

3. Reveille, J. D. Epidemiology of spondyloarthritis in North America. *Am. J. Med. Sci.* **341,** 284–286 (2011).

4. Neuhausen, S. L. *et al.* Co-occurrence of celiac disease and other autoimmune diseases in celiacs and their first-degree relatives. *J. Autoimmun.* **31,** 160–165 (2008).

5. Ludvigsson, J. F., Lindelöf, B., Zingone, F. & Ciacci, C. Psoriasis in a nationwide cohort study of patients with celiac disease. *J. Invest. Dermatol.* **131,** 2010–2016 (2011).

6. Gelfand, J. M. *et al.* The prevalence of psoriasis in African Americans: Results from a population-based study. *J. Am. Acad. Dermatol.* **52,** 23–6 (2005).

7. Augustin, M. *et al.* Epidemiology and comorbidity of psoriasis in children. *Br. J. Dermatol.* **162,** 633–636 (2010).

8. Iqbal, T., Zaidi, M. A., Wells, G. A. & Karsh, J. Celiac disease arthropathy and autoimmunity study. *J. Gastroenterol. Hepatol.* **28,** 99–105 (2013).

9. Sategna-Guidetti, C. *et al.* Prevalence of thyroid disorders in untreated adult celiac disease patients and effect of gluten withdrawal: An Italian multicenter study. *Am. J. Gastroenterol.* **96,** 751–757 (2001).

10. Ludvigsson, J. J. F., Ludvigsson, J. J. F., Ekbom, A. & Montgomery, S. M. Celiac disease and risk of subsequent type 1 diabetes: A general population cohort study of children and adolescents. *Diabetes Care* **29,** 2483–2488 (2006).

11. Corporaal, S., Bijl, M. & Kallenberg, C. G. Familial occurrence of autoimmune diseases and autoantibodies in a Caucasian population of patients with systemic lupus erythematosus. *Clin Rheumatol* **21,** 108–113 (2002).

12. Bernatsky, S. *et al.* A population-based assessment of systemic lupus erythematosus incidence and prevalence--results and implications of using administrative data for epidemiological studies. *Rheumatology (Oxford).* **46,** 1814–1818 (2007).

13. Boyle, J. M. & Buckley, R. H. Population prevalence of diagnosed primary immunodeficiency diseases in the United States. *J. Clin. Immunol.* **27,** 497–502 (2007).

14. Deane, S., Selmi, C., Naguwa, S. M., Teuber, S. S. & Gershwin, M. E. Common variable immunodeficiency: Etiological and treatment issues. *Int. Arch. Allergy Immunol.* **150,** 311–324 (2009).

15. Myrtek, D. & Salzer, U. Common Variable Immunodeficiency Genetic INsights into a Complex and Baffling Disease. *Rheumatol.* (1998).

16. Weng, X., Liu, L., Barcellos, L. F., Allison, J. E. & Herrinton, L. J. Clustering of inflammatory bowel disease with immune mediated diseases among members of a northern California-managed care organization. *Am. J. Gastroenterol.* **102,** 1429–1435 (2007).

17. Loftus, E. V *et al.* Ulcerative colitis in Olmsted County, Minnesota, 1940-1993: incidence, prevalence, and survival. *Gut* **46,** 336–343 (2000).

18. Nielsen, N. M. *et al.* Type 1 diabetes and multiple sclerosis: A Danish population-based cohort study. *Arch. Neurol.* **63,** 1001–1004 (2006).

19. Greco, D., Pisciotta, M., Gambina, F. & Maggio, F. Celiac disease in subjects with type 1 diabetes mellitus: A prevalence study in western Sicily (Italy). *Endocrine* **43,** 108–111 (2013).

20. Prahalad, S. *et al.* Familial aggregation of juvenile idiopathic arthritis. *Arthritis Rheum.* **50,** 4022–4027 (2004).

21. Helmick, C. G. *et al.* Estimates of the prevalence of arthritis and other rheumatic conditions in the United States. Part I. *Arthritis Rheum.* **58,** 15–25 (2008).

22. Von Koskull, S., Truckenbrodt, H., Holle, R. & Hörmann, A. Incidence and prevalence of juvenile arthritis in an urban population of southern Germany: a prospective study. *Ann. Rheum. Dis.* **60,** 940–945 (2001).

23. Turkcapar, N. *et al.* The prevalence of extraintestinal manifestations and HLA association in patients with inflammatory bowel disease. *Rheumatol. Int.* **26,** 663–668 (2006).

24. Halfvarson, J., Bodin, L., Tysk, C., Lindberg, E. & Järnerot, G. Inflammatory bowel disease in a Swedish twin cohort: A long- term follow-up of concordance and clinical characteristics. *Gastroenterology* **124,** 1767–1773 (2003).

25. Orholm, M., Binder, V., Sørensen, T. I., Rasmussen, L. P. & Kyvik, K. O. Concordance of inflammatory bowel disease among Danish twins. Results of a nationwide study. *Scand. J. Gastroenterol.* **35,** 1075–1081 (2000).

26. Mearin, M. L. Celiac disease among children and adolescents. *Curr. Probl. Pediatr. Adolesc. Health Care* **37,** 86–105 (2007).

27. Polanko, I., Biemond, I. & Van Leeuwen, A. in *Genet. coeliac Dis.* (ed. McConnell, R.) 211–234 (MTP Press, 1981).

28. Kamath, K. & Dorney, S. Is discordance for coeliac disease in monozygotic twins permanent? *Pediatr. Res.* **17,** (1983).

29. Kaprio, J. *et al.* Concordance for type 1 (insulin-dependent) and type 2 (non-insulin-dependent) diabetes mellitus in a population-based cohort of twins in Finland. *Diabetologia* **35,** 1060–1067 (1992).

30. Prahalad, S. Genetic analysis of juvenile rheumatoid arthritis: approaches to complex traits. *Curr. Probl. Pediatr. Adolesc. Health Care* **36,** 83–90 (2006).

31. Lønnberg, A. S. *et al.* Heritability of psoriasis in a large twin sample. *Br. J. Dermatol.* **169,** 412–416 (2013).

32. Elder, J. T. *et al.* The genetics of psoriasis. *Arch. Dermatol.* **130,** 216–224 (1994).

33. Block, S. R., Winfield, J. B., Lockshin, M. D., D'Angelo, W. A. & Christian, C. L. Studies of twins with systemic lupus erythematosus. A review of the literature and presentation of 12 additional sets. *Am. J. Med.* **59,** 533–552 (1975).

34. Deapen, D. *et al.* A revised estimate of twin concordance in systemic lupus erythematosus. *Arthritis Rheum.* **35,** 311–318 (1992).

35. Järvinen, P. Occurrence of ankylosing spondylitis in a nationwide series of twins. *Arthritis Rheum.* **38,** 381–383 (1995).

36. Hamersma, J. *et al.* Is disease severity in ankylosing spondylitis genetically determined? *Arthritis Rheum.* **44,** 1396–1400 (2001).

37. Mittag, F. *et al.* Use of support vector machines for disease risk prediction in genome-wide association studies: Concerns and opportunities. *Hum. Mutat.* **33,** 1708–1718 (2012).

38. Hyttinen, V., Kaprio, J., Kinnunen, L., Koskenvuo, M. & Tuomilehto, J. Genetic liability of type 1 diabetes and the onset age among 22,650 young Finnish twin pairs: a nationwide follow-up study. *Diabetes* **52,** 1052–1055 (2003).

39. So, H.-C., Gui, A. H. S., Cherny, S. S. & Sham, P. C. Evaluating the heritability explained by known susceptibility variants: a survey of ten complex diseases. *Genet. Epidemiol.* **35,** 310–7 (2011).

40. Zaitlen, N. *et al.* Using extended genealogy to estimate components of heritability for 23 quantitative and dichotomous traits. *PLoS Genet.* **9,** e1003520 (2013).

41. Lee, S. H. *et al.* Estimation of SNP heritability from dense genotype data. *Am. J. Hum. Genet.* **93,** 1151–5 (2013).

42. Chen, G.-B. *et al.* Estimation and partitioning of (co)heritability of inflammatory bowel disease from GWAS and immunochip data. *Hum. Mol. Genet.* (2014). doi:10.1093/hmg/ddu174

43. Lee, S. H., Yang, J., Goddard, M. E., Visscher, P. M. & Wray, N. R. Estimation of pleiotropy between complex diseases using single-nucleotide polymorphism-derived genomic relationships and restricted maximum likelihood. *Bioinformatics* **28,** 2540–2 (2012).

44. Speed, D., Hemani, G., Johnson, M. R. & Balding, D. J. Improved heritability estimation from genome-wide SNPs. *Am. J. Hum. Genet.* **91,** 1011–21 (2012).

45. Speed, D. & Balding, D. J. MultiBLUP: improved SNP-based prediction for complex traits. *Genome Res.* gr.169375.113– (2014). doi:10.1101/gr.169375.113

46. Jia, X. *et al. Imputing Amino Acid Polymorphisms in Human Leukocyte Antigens*. *PLoS One* **8,** (Public Library of Science, 2013).

47. Zhu, K.-J. *et al.* Psoriasis regression analysis of MHC loci identifies shared genetic variants with vitiligo. *PLoS One* **6,** e23089 (2011).

48. Darren, C., Robins, G. & Howdle, P. D. Advances in Celiac Disease. *Curr Opin Gastroenterol* **23,** 142–148 (2007).

49. Sollid, L. M. & Jabri, B. Triggers and drivers of autoimmunity: lessons from coeliac disease. *Nat. Rev. Immunol.* **13,** 294–302 (2013).

50. Guerra, S. G., Vyse, T. J. & Cunninghame Graham, D. S. The genetics of lupus: a functional perspective. *Arthritis Res. Ther.* **14,** 211 (2012).

51. Fernando, M. M. A. *et al.* Defining the role of the MHC in autoimmunity: A review and pooled analysis. *PLoS Genet.* **4,** e1000024 (2008).

52. Burton, P. R. *et al.* Association scan of 14,500 nonsynonymous SNPs in four diseases identifies autoimmunity variants. *Nat. Genet.* **39,** 1329–1337 (2007).

53. Munthe-Kaas, M. C. *et al.* HLA Dr-Dq haplotypes and the TNFA-308 polymorphism: Associations with asthma and allergy. *Allergy Eur. J. Allergy Clin. Immunol.* **62,** 991–998 (2007).

54. Jacobson, E. M., Huber, A., Tomer, Y. & Y, T. The HLA gene complex in thyroid autoimmunity: From epidemiology to etiology. *J. Autoimmun.* **30,** 58–62 (2008).

55. Hunt, K. A. *et al.* Newly identified genetic risk variants for celiac disease related to the immune response. *Nat. Genet.* **40,** 395–402 (2008).

56. Van Heel, D. A. *et al.* A genome-wide association study for celiac disease identifies risk variants in the region harboring IL2 and IL21. *Nat. Genet.* **39,** 827–829 (2007).

57. Barrett, J. C. *et al.* Genome-wide association defines more than 30 distinct susceptibility loci for Crohn's disease. *Nat. Genet.* **40,** 955–962 (2008).

58. Hafler, D. A. *et al.* Risk alleles for multiple sclerosis identified by a genomewide study. *N. Engl. J. Med.* **357,** 851–862 (2007).

59. Consortium, I. M. S. G. *et al.* Genetic risk and a primary role for cell-mediated immune mechanisms in multiple sclerosis. *Nature* **476,** 214 (2011).

60. S, D. *et al.* A genome-wide association study of psoriasis and psoriatic arthritis identifies new disease loci. *PLoS Genet.* **4,** e1000041 (2008).

61. Bowes, J. & Barton, A. Recent advances in the genetics of RA susceptibility. *Rheumatology* **47,** 399–402 (2008).

62. Raychaudhuri, S. *et al.* Common variants at CD40 and other loci confer risk of rheumatoid arthritis. *Nat. Genet.* **40,** 1216–1223 (2008).

63. Harley, J. B. *et al.* Genome-wide association scan in women with systemic lupus erythematosus identifies susceptibility variants in ITGAM, PXK, KIAA1542 and other loci. *Nat. Genet.* **40,** 204–210 (2008).

64. P, D. *et al.* Genome-wide association study of 14 , 000 cases of seven common diseases and. *Nature* **447,** 661–678 (2007).

65. Fisher, S. A. *et al.* Genetic determinants of ulcerative colitis include the ECM1 locus and five loci implicated in Crohn's disease. *Nat. Genet.* **40,** 710–712 (2008).

66. Franke, A. *et al.* Sequence variants in IL10, ARPC2 and multiple other loci contribute to ulcerative colitis susceptibility. *Nat. Genet.* **40,** 1319–1323 (2008).

67. M, A. Sarcoidosis is associated with a truncating splice site mutation in BTNL2. *Nat. Genet.* **37,** 357 (2005).

68. Abolhassani, H. *et al.* Autoimmune phenotype in patients with common variable immunodeficiency. *J. Investig. Allergol. Clin. Immunol.* **23,** 323–9 (2013).

69. Boileau, J. *et al.* Autoimmunity in common variable immunodeficiency: Correlation with lymphocyte phenotype in the French DEFI study. *J. Autoimmun.* **36,** 25–32 (2011).

70. Robazzi, T. C. *et al.* Autoimmune endocrine disorders and coeliac disease in children and adolescents with juvenile idiopathic arthritis and rheumatic fever. *Clin. Exp. Rheumatol.* **31,** 0310–0317 (2013).

71. Stagi, S., Giani, T., Simonini, G. & Falcini, F. Thyroid function, autoimmune thyroiditis and coeliac disease in juvenile idiopathic arthritis. *Rheumatology (Oxford).* **44,** 517–520 (2005).

72. Eaton, W. W., Rose, N. R., Kalaydjian, A., Pedersen, M. G. & Mortensen, P. B. Epidemiology of autoimmune diseases in Denmark. *J. Autoimmun.* **29,** 1–9 (2007).

73. Li, W.-Q., Qureshi, A. A., Schernhammer, E. S. & Han, J. Rotating night-shift work and risk of psoriasis in US women. *J. Invest. Dermatol.* **133,** 565–7 (2013).

74. Cohen, A. D., Dreiher, J. & Birkenfeld, S. Psoriasis associated with ulcerative colitis and Crohn's disease. *J. Eur. Acad. Dermatol. Venereol.* **23,** 561–565 (2009).

75. Birkenfeld, S., Dreiher, J., Weitzman, D. & Cohen, A. D. Coeliac disease associated with psoriasis. *Br. J. Dermatol.* **161,** 1331–1334 (2009).

76. Somers, E. C., Thomas, S. L., Smeeth, L. & Hall, A. J. Are individuals with an autoimmune disease at higher risk of a second autoimmune disorder? *Am. J. Epidemiol.* **169,** 749–755 (2009).

77. Mollazadegan, K. *et al.* A population-based study of the risk of diabetic retinopathy in patients with type 1 diabetes and celiac disease. *Diabetes Care* **36,** 316–321 (2013).

78. Sanchez-Albisua, I. *et al.* Coeliac disease in children with Type 1 diabetes mellitus: the effect of the gluten-free diet. *Diabet. Med.* **22,** 1079–1082 (2005).

79. Li Voon Chong, J. S. W., Leong, K. S., Wallymahmed, M., Sturgess, R. & MacFarlane, I. A. Is coeliac disease more prevalent in young adults with coexisting Type 1 diabetes mellitus and autoimmune thyroid disease compared with those with Type 1 diabetes mellitus alone? *Diabet. Med.* **19,** 334–7 (2002).

80. Boelaert, K. *et al.* Prevalence and Relative Risk of Other Autoimmune Diseases in Subjects with Autoimmune Thyroid Disease. *Am. J. Med.* **123,** (2010).

81. Orchard, T. R., Wordsworth, B. P. & Jewell, D. P. Peripheral arthropathies in inflammatory bowel disease: their articular distribution and natural history. *Gut* **42,** 387–391 (1998).

82. Palm, O., Moum, B., Ongre, A. & Gran, J. T. Prevalence of ankylosing spondylitis and other spondyloarthropathies among patients with inflammatory bowel disease: a population study (the IBSEN study). *J. Rheumatol.* **29,** 511–515 (2002).

83. Gupta, G., Gelfand, J. M. & Lewis, J. D. Increased risk for demyelinating diseases in patients with inflammatory bowel disease. *Gastroenterology* **129,** 819–826 (2005).
